# A humanized chemogenetic system inhibits murine pain-related behavior and hyperactivity in human sensory neurons

**DOI:** 10.1126/scitranslmed.adh3839

**Published:** 2023-10-04

**Authors:** Jimena Perez-Sanchez, Steven J. Middleton, Luke A. Pattison, Helen Hilton, Mosab Ali Awadelkareem, Sana R. Zuberi, Maria B. Renke, Huimin Hu, Xun Yang, Alex J. Clark, Ewan St. John Smith, David L. Bennett

**Affiliations:** 1 Nuffield Department of Clinical Neurosciences, University of Oxford; Oxford OX3 9DU, UK; 2 Department of Pharmacology, University of Cambridge; Cambridge CB2 1PD, UK; 3 Blizard Institute, Barts and the London School of Medicine and Dentistry; London E1 2AT, UK

## Abstract

Hyperexcitability in sensory neurons is known to underlie many of the maladaptive changes associated with persistent pain. Chemogenetics has shown promise as a means to suppress such excitability, yet chemogenetic approaches suitable for human applications are needed. PSAM^4^-GlyR is a modular system based on the human α7 nicotinic acetylcholine and glycine receptors, which responds to inert chemical ligands and the clinically approved drug, varenicline. Here, we demonstrated the efficacy of this channel in silencing both mouse and human sensory neurons by the activation of large shunting conductances after agonist administration. Virally mediated expression of PSAM^4^-GlyR in mouse sensory neurons produced behavioral hyposensitivity upon agonist administration, which was recovered upon agonist washout. Importantly, stable expression of the channel led to similar reversible suppression of pain related behaviour even after 10 months of viral delivery. Mechanical and spontaneous pain readouts were also ameliorated by PSAM^4^-GlyR activation in acute and joint pain inflammation mouse models. Furthermore, suppression of mechanical hypersensitivity generated by a spared nerve injury model of neuropathic pain was also observed upon activation of the channel. Effective silencing of behavioural hypersensitivity was reproduced in a human model of hyperexcitability and clinical pain: PSAM^4^-GlyR activation decreased the excitability of human induced pluripotent stem-cell-derived sensory neurons and spontaneous activity due to a gain of function Na_V_1.7 mutation causing inherited erythromelalgia. Our results demonstrate the contribution of sensory neuron hyperexcitability to neuropathic pain and the translational potential of an effective, stable and reversible humanized chemogenetic system for the treatment of pain.

## Introduction

Chronic pain remains a critical unmet clinical challenge. Approximately one in five adults suffers from chronic pain (1), which can arise as a consequence of tissue damage, disease or inflammation (2, 3). Current treatments have poor efficacy and tolerability (4); the devastating opioid crisis highlights the need for new, more effective, non-addictive treatments for this condition. However, the varied and complex pathophysiological mechanisms that underlie chronic pain have made this a particularly difficult task.

Increased activity in sensory neurons, which relay information from the periphery to the central nervous system (CNS), has been identified as a common driver for the induction and maintenance of inflammatory (5) and neuropathic pain following tissue injury (6–8). Sensory neurons rely on the generation of action potentials (APs) to encode such information. Increased AP spiking (hyperexcitability) is a hallmark of altered sensory neuron processing, observed as changes in firing pattern, bursting and the generation of spontaneous activity (9–15). Intrinsic excitability arises from the complex interaction of multiple ion channels (16), which means that countless molecular changes may be responsible for producing altered activity in different chronic pain conditions. Extrinsic factors such as immune mediators can also sensitize sensory neurons (17). So far, efforts to ascribe single molecular changes to altered activity have made headway to recognize potential therapeutic targets, but they have not necessarily been translated successfully into treatments (4, 18, 19). An alternative plan to guide future strategies is to surpass the molecular mechanisms that enhance sensory neuron excitability and directly suppress the activity in these neurons. Indeed, local anesthetics that block the generation of APs in peripheral nerves transiently reduce both spontaneous and evoked pain in animal models of nerve injury, as well as in patients with neuropathic and osteoarthritis pain(8, 20–22). Nevertheless, such non-specific approaches are not clinically viable, because systemic administration may give rise to motor, cardiac and CNS side-effects (4).

Chemogenetic approaches serve this purpose as they can modulate neuronal activity in defined neuronal populations, by expressing designer receptors that respond selectively to specific agonists which enables dose dependent control and reversibility (23). Ideally, these approaches should be compatible with human tissue to provide potential for gene therapy. Indeed, chemogenetic approaches have been used to suppress the activity of sensory neurons and pain signaling (24–27). However, several concerns have been raised regarding agonist viability of the most common chemogenetic tools, as well as their disruption of endogenous receptor function (26, 28, 29). Furthermore, chemogenetic strategies to suppress ectopic activity have never been used in human models of neuropathic pain. The recently designed pharmacologically-selective actuator modules (PSAMs) are promising in this regard (30, 31). They are modular systems based on a modified human ɑ7 nicotinic acetylcholine (ACh) receptor ligand-binding domain, which does not bind ACh, but ultrapotent pharmacologically-selective effector molecules (uPSEMs) and in the case of the most recent version PSAM^4^, the FDA-approved drug varenicline. This, in combination with the human glycine receptor pore domain, gives rise to PSAM^4^-GlyR, a chloride channel activated by selective agonists. This tool has been used to inhibit neuronal activity in the brain, although its inhibitory effect is highly dependent on the chloride gradient (31, 32). In this study, we used PSAM^4^-GlyR to silence the activity of mouse sensory neurons in acute, inflammatory and neuropathic pain conditions. Furthermore, to evaluate its potential for human therapeutic application, we expressed PSAM^4^-GlyR in human sensory neurons, where we used it to silence neuronal activity, as well as ectopic activity in a human cellular model of neuropathic pain.

## Results

### Generation of a new PSAM^4^-GlyR construct

**Fig. 1:**
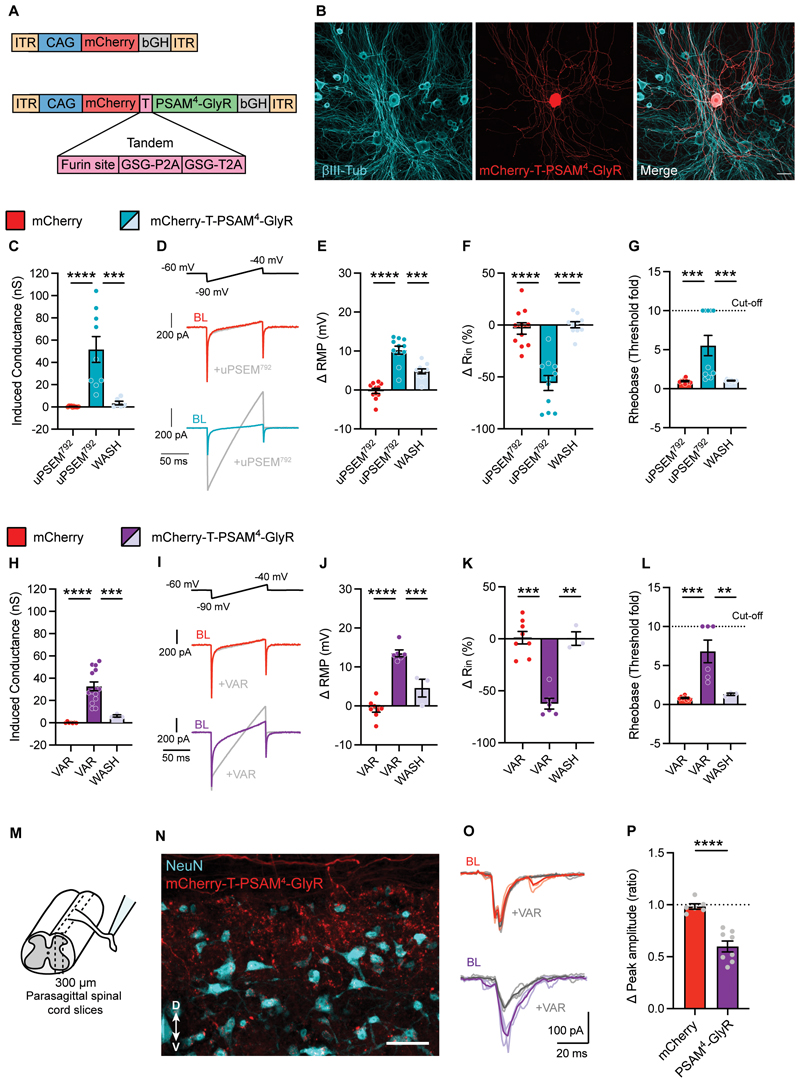
PSAM^4^-GlyR can reversibly silence mouse sensory neurons and reduce synaptic transmission. **(A)** Schematic representation of the constructs used in this study: mCherry and mCherry-T-PSAM^4^-GlyR. **(B)** Example images of dissociated sensory neurons transduced with mCherry-T-PSAM^4^-GlyR after 2 days in vitro (Scale bar = 50 μm). **(C)** Voltage-clamp recordings of membrane conductance in the presence of uPSEM^792^ (10 nM) and after agonist washout (mCherry: n = 10 cells; mCherry-T-PSAM^4^-GlyR: n = 9 cells). (**D**) Representative current traces obtained by the application of voltage ramps used to determine membrane conductance. BL: Baseline. **(E-G)** Change in resting membrane potential (RMP) **(E)** input resistance (Rin) **(F)** and rheobase **(G)** after uPSEM^792^ administration and washout. The cut-off rheobase value was defined as 10 times the threshold rheobase before uPSEM^792^ treatment. (mCherry: n = 11 cells; mCherry-T-PSAM^4^-GlyR: n = 11 cells). **(H)** Membrane conductance measurements in PSAM^4^-GlyR neurons after varenicline (VAR; 20 nM) administration and agonist washout (mCherry: n = 7 cells; mCherry-T-PSAM^4^-GlyR: n = 14 cells). (**I**) Representative current traces obtained by the application of voltage ramps used to determine membrane conductance. **(J–L)** Changes in resting membrane potential (RMP) **(J)** input resistance **(K)** and rheobase **(L)** after varenicline administration and washout (mCherry: n = 8 cells; mCherry-T-PSAM^4^-GlyR: n = 6 cells). **C-L:** One-way ANOVA with Tukey Post-hoc, ** P < 0.01, *** P < 0.001, **** P < 0.0001). **(M)** Schematic representation of the experimental procedure for spinal cord slices. **(N)** Example image of a parasagittal slice used for recording, showing mCherry-T-PSAM^4^-GlyR expressing terminals in the dorsal horn of the spinal cord. D: dorsal; V: ventral (Scale bar = 50 μm). **(O)** Representative postsynaptic currents evoked by dorsal root stimulation (ePSCs) recorded from superficial dorsal horn neurons obtained from mCherry-transduced animals (red) or mCherry-T-PSAM^4^-GlyR animals (purple) before and after application of varenicline (20 nM; grey). **(P)** Change in ePSC amplitude after application of varenicline (mCherry: n = 6 cells; mCherry-T-PSAM^4^-GlyR: n = 8 cells. Unpaired t-test, **** P<0.0001). All data are expressed as mean ± S.E.M.

We had to consider several factors when designing a PSAM^4^-GlyR construct for use in sensory neurons. We required a strong promotor, a red fluorescent tag, and a short, efficient linker. We modified the original construct containing PSAM^4^-GlyR (31) to account for these factors. We also ensured its size was reduced, suitable for packaging into adeno-associated virus (AAV) vectors (33, 34), in preparation for in vivo delivery to sensory neurons. After generating several candidates, we found the most promising construct to be pCAG-mCherry-tPT2A-PSAM4-GlyR (mCherry-T-PSAM^4^-GlyR) (see Methods). We assessed expression and function of our construct in HEK293t cells and used whole-cell patch clamp recordings to measure the induced membrane conductance following addition of varenicline, the clinically relevant agonist of PSAM^4^-GlyR (31). We confirmed that mCherry-T-PSAM^4^-GlyR was functional, and observed a large increase in membrane conductance following varenicline application, similar to the original unmodified construct (Fig. S1). We therefore selected mCherry-T-PSAM^4^-GlyR (and mCherry-alone to use as control) for all ensuing experiments (Fig. 1A).

### Activation of PSAM^4^-GlyR silences electrical activity of mouse sensory neurons

To assess the suitability of PSAM^4^-GlyR as a chemogenetic silencer in sensory neurons, we transfected dorsal root ganglion (DRG) sensory neurons acutely dissociated from mice with mCherry-alone or mCherry-T-PSAM^4^-GlyR constructs (Fig. 1B), and performed fluorescence targeted whole-cell patch clamp recordings. To determine the optimal concentration of agonists (uPSEM^792^ or varenicline) to activate PSAM^4^-GlyR in in vitro experiments, we measured changes in membrane conductance at different agonist doses (Fig. S2A-B). Administration the ultrapotent designer agonist uPSEM^792^ at 2 nM produced a small increase in membrane conductance which did not reach significance (8.9 ± 3.5 nS; p=0.2). However, progressively increasing uPSEM^792^ concentration increased membrane conductance at 10 nM (38.7 ± 7.4 nS), 20 nM (43.4 ± 8.6 nS) and 100 nM (42.3 ± 8.3 nS) compared to mCherry control neurons (Fig. S2A). For all further in vitro experiments, we selected 10 nM uPSEM^792^ as appropriate for PSAM^4^-GlyR activation. As in cortical neurons (31), very low concentrations of varenicline were required to increase membrane conductance in PSAM^4^-GlyR-expressing cells. We found that administration of 2 nM varenicline did not produce a significant increase in membrane conductance (3.7 ± 1.5 nS; P < 0.05) compared to control neurons expressing only mCherry, but increasing the dose to 10 nM increased the membrane conductance to 27.8 ± 4.8 nS which was significantly different from mCherry control neurons (p = 0.0005) (Fig. S2B). The conductance was not increased further with 20 nM (29.1 ± 4.1 nS;) or 100 nM (24.2 ± 4.7 nS) varenicline (Fig. S2B). We therefore decided to use 20 nM varenicline in all subsequent experiments to ensure sufficient PSAM^4^-GlyR activation.

We next addressed whether such an increase in membrane conductance impacts sensory neuron excitability. Sensory neurons were recorded in whole-cell current-clamp configuration, with a chloride load in the recording pipette (30 mM) to mimic the high intracellular chloride concentration maintained by sensory neurons (35, 36). Under these conditions, activation of PSAM^4^-GlyR with uPSEM^792^, shown as an increase in membrane conductance (Fig. 1C-D) resulted in membrane potential (*V*_m_) depolarization (Fig. 1E), according to the chloride reversal potential estimated with the Nernst equation (*E_Cl_* = -41 mV). Depolarization of *V*_m_ by agonist administration in PSAM^4^-GlyR neurons did not produce any AP firing (Fig. S3A-B), but was efficient in silencing sensory neurons via a decrease in input resistance, compared to mCherry control neurons (Fig. 1F). Furthermore, agonist administration increased the current required to generate an AP (rheobase) in PSAM^4^-GlyR expressing neurons, but not in control neurons (Fig. 1G). Higher agonist concentrations increased the degree and the rate of depolarization, but still failed to produce any AP firing (Fig. S3C-F). Activation of PSAM4-GlyR with varenicline showed a similar increase in membrane conductance (Fig. 1H-I), *V*_m_ depolarization (Fig. 1J), decrease in input resistance (Fig. 1K) and increase in rheobase (Fig.1L). All the effects of agonist induced PSAM^4^-GlyR silencing were reversible in vitro; we observed washout and return to baseline of all parameters after 15 mins of agonist wash (Fig 1C-L). These results suggest that activation of PSAM^4^-GlyR potently, effectively and reversibly silences sensory neuron activity through large shunting chloride conductances.

### PSAM^4^-GlyR activation suppresses synaptic transmission to dorsal horn neurons

To determine whether silencing of neuronal activity observed at the soma translates into effective silencing at the presynaptic terminals in the spinal cord, we measured synaptic transmission in a parasagittal spinal cord slice preparation. To ease delivery of the construct in vivo, we generated AAV serotype 9 particles containing either mCherry-alone or mCherry-T-PSAM^4^-GlyR. Spinal cord slices were obtained from virally transduced mice that had received neonatal subcutaneous AAV (Fig. S4A). This strategy targets the peripheral nervous system (Fig. S4B-E) but not spinal cord neurons (Fig. S4F) (37). Whole-cell patch clamp recordings were obtained from lamina II dorsal horn neurons and monosynaptic excitatory post-synaptic currents evoked by electric stimulation of dorsal roots (eEPSCs) were measured (Fig. 1M-N). Superfusion of varenicline in spinal cord slices did not change the magnitude of eEPSCs in control neurons obtained from mCherry-transduced animals, whereas it decreased the magnitude of postsynaptic responses obtained from neurons from PSAM^4^-GlyR expressing animals (Fig. 1O-P). This suggests that the silencing of electric activity at the soma and central terminals of sensory neurons effectively interferes with the relay of information to the spinal cord.

### Activation of PSAM^4^-GlyR leads to decreased thermal and mechanical sensitivity

Once we determined the neuronal silencing capacity of PSAM^4^-GlyR activation in vitro and ex vivo, we then explored whether PSAM^4^-GlyR activation silences sensory behaviors in vivo. We virally delivered PSAM^4^-GlyR by intrathecal (i.t.) injection to selectively transduce sensory neurons in the DRG, while avoiding transduction of neurons in the spinal cord, which is physically separated by meningeal membranes (38). 6-8 weeks post i.t. injection we measured the effect of PSAM^4^-GlyR activation upon thermal sensitivity using the Hargreaves radiant heat source (Fig. S5A). We established a time course after an intraperitoneal (i.p.) injection of uPSEM^792^ (1mg/kg), where we saw a decrease in thermal sensitivity at 2 hours from treatment which returned to baseline after 5 hours (Fig. S5B, left). One week after uPSEM^792^ administration, thermal thresholds were comparable to baseline which enabled reactivation of PSAM^4^-GlyR by subsequent administrations (Fig. S5B, right). Treatment with a higher dose of uPSEM^792^ (5mg/kg) produced a stronger decrease in thermal sensitivity after 1 hour, again peaking at 2 hours from i.p. injection, which recovered after 5 hours (Fig. S5C-D). From these data we were confident that our silencing system was functional in vivo.

**Fig. 2:**
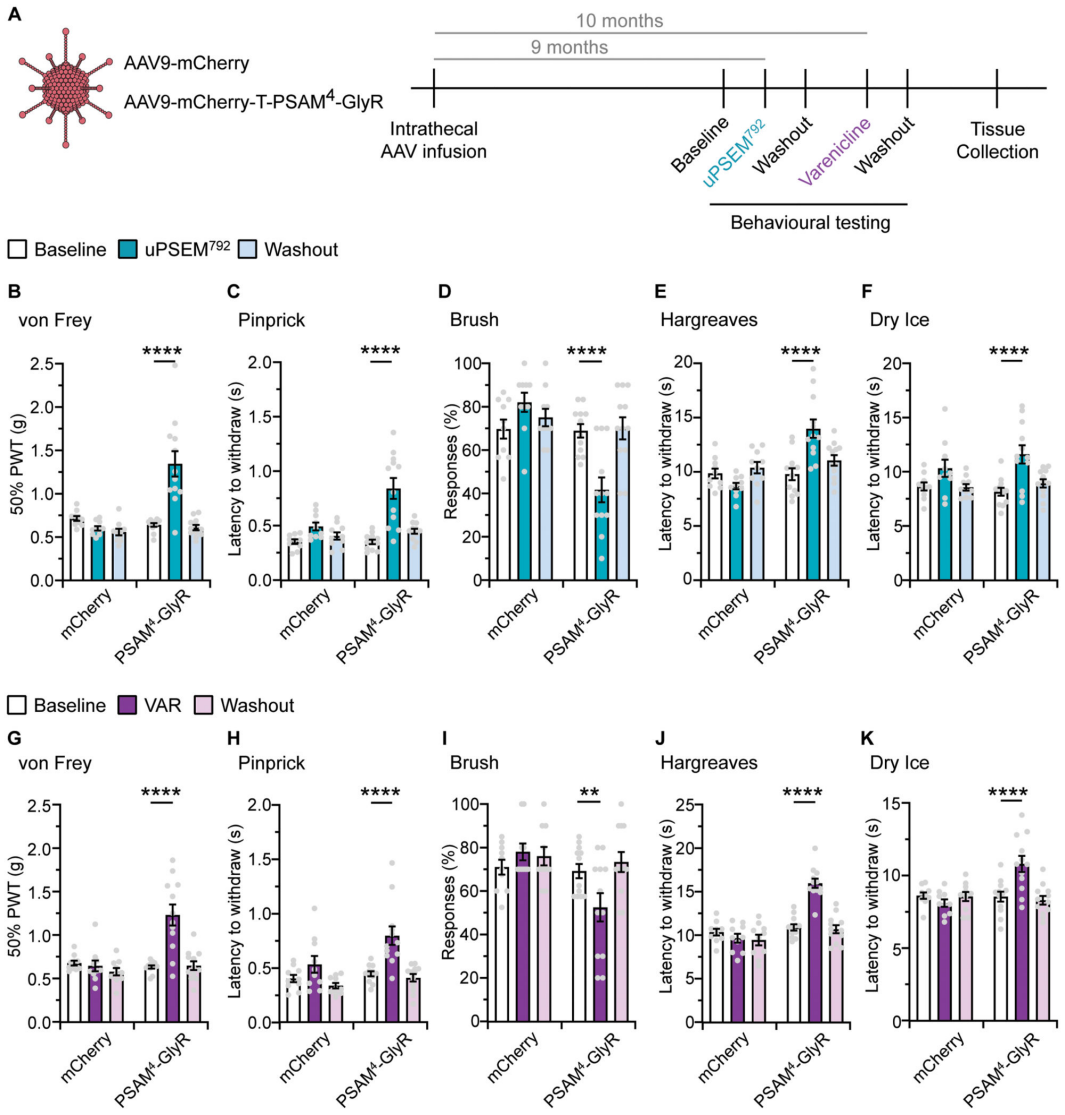
Robust, reversible, and repeatable silencing of acute sensory behaviors via agonist-induced activation of PSAM^4^-GlyR. (**A**) Timeline of the experimental design. Intrathecal infusion of AAV-mCherry or mCherry-T-PSAM^4^-GlyR followed by baseline sensory testing, and re-testing during or post uPSEM^792^ or varenicline. (**B–D**) Mechanical sensory testing in mCherry or PSAM^4^-GlyR expressing mice pre (baseline), during and post (washout) uPSEM^792^; von Frey (**B**), Pinprick (**C**) and Brush (**D**). (**E** and **F**) Thermal sensory in mCherry or PSAM^4^-GlyR expressing mice pre (baseline), during and post (washout) uPSEM^792^; Hargreaves (**E**) and Dry Ice (**F**). (**G–I**) Mechanical sensory testing in mCherry or PSAM^4^-GlyR expressing mice pre (baseline), during and post (washout) varenicline; von Frey (**G**), Pinprick (**H**) and Brush (**I**). (**J** and **K**) Thermal sensory testing in mCherry or PSAM^4^-GlyR expressing mice pre (baseline), during and post (washout) varenicline; Hargreaves (**J**) and Dry Ice (**K**) (mCherry: n = 10 mice, PSAM^4^-GlyR: n = 12 mice, all data sets RM-two way ANOVA, post-hoc Bonferroni test, ** P < 0.01, **** P < 0.0001). Data expressed as mean ± S.E.M.

From a clinical translation perspective, the effective application of gene therapy relies on long-term expression of the transgene. To look at the stability of PSAM^4^-GlyR expression over time, we tested again the same behavioral cohort 9-10 months after viral delivery. Following uPSEM^792^ administration (5 mg/kg), PSAM^4^-GlyR expressing mice became hyposensitive to punctate mechanical stimuli (von Frey; Fig.2B), noxious mechanosensation (pinprick; Fig. 2C.) and light touch (brush; Fig. 2D). In addition, mice also became hyposensitive to noxious hot and cold thermal stimuli (Hargreaves; Fig. 2E. and dry ice; Fig. 2F) after treatment with uPSEM^792^. Control mice that received mCherry-alone were unaffected by uPSEM^792^ administration in all assays. Complete washout of uPSEM^792^ was achieved and all changes in sensory sensitivity returned to baseline (Fig. 2B-F). 1-month later, reactivation of PSAM^4^-GlyR with varenicline (0.3mg/kg), produced a similar behavioral hyposensitivity to sensory stimuli throughout all assays in PSAM^4^-GlyR expressing mice, which was completely reversible (Fig 2G-K). The same dose of varenicline had no effects on the sensory function of control mice (Fig. 2G-K). In contrast, varenicline administration produced no proprioceptive changes in PSAM^4^-GlyR expressing mice, as they behaved similar to control mice during the beam task (Fig. S6A-D). Taken together, PSAM^4^-GlyR expression and function remained stable in the long-term and yielded reversible silencing of sensory behaviors upon activation with either the designer or the clinically viable PSAM^4^-GlyR agonist.

### Stable expression of PSAM^4^-GlyR in DRG sensory neurons

**Fig. 3:**
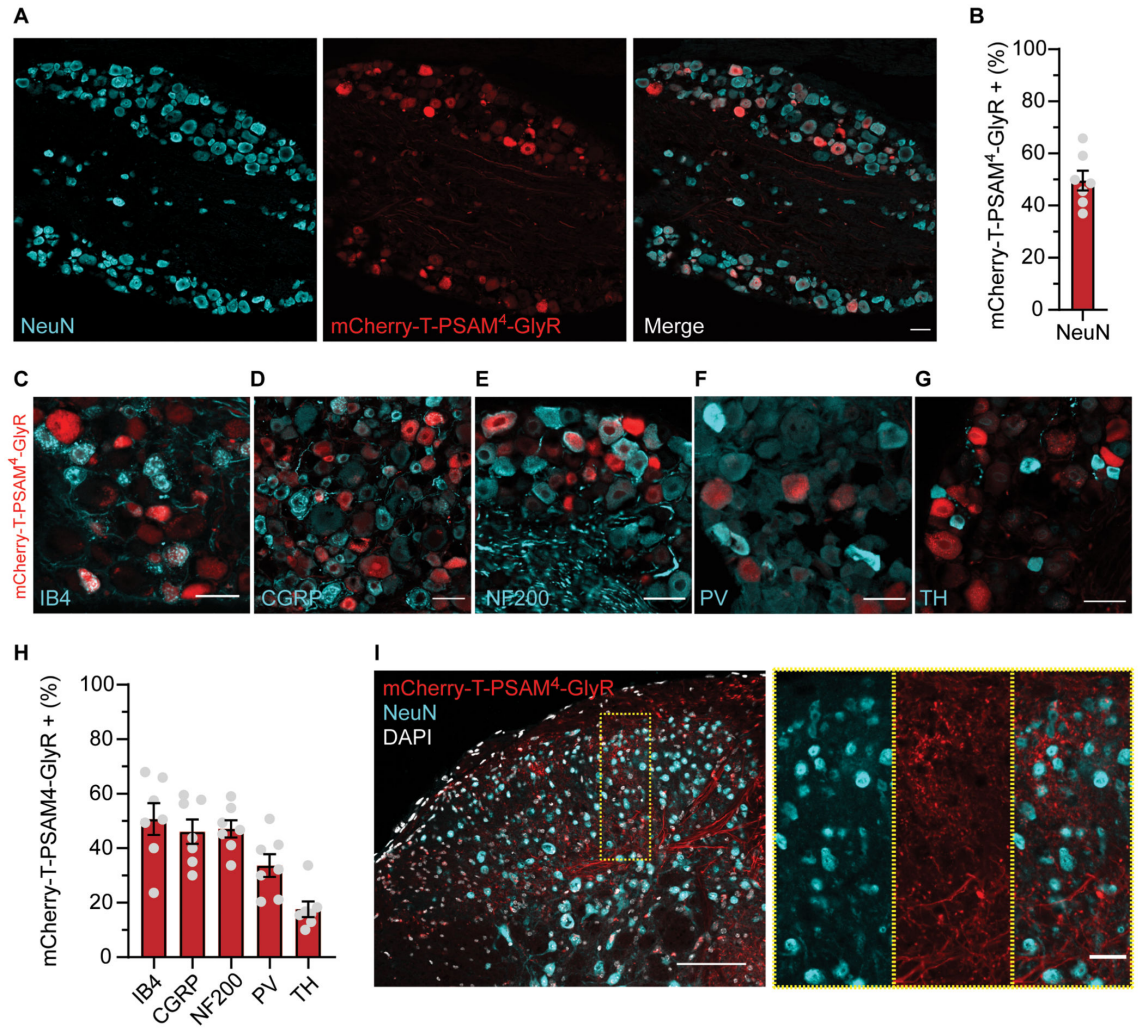
Long-term, stable expression of AAV9-mCherry-T-PSAM^4^-GlyR selectively in sensory neurons. (**A**) Example image of DRG neurons transduced by AAV9-mCherry-T-PSAM^4^-GlyR 11-months post i.t. injection (Scale bar 50 um) (**B**) Quantification of NeuN positive neurons that are also mCherry-T-PSAM^4^-GlyR positive (n = 7 mice, 1631/3229 neurons) (**C–G**) Example images of DRG neuron subpopulation markers, isolectin-B4 (IB4) (**C**), calcitonin gene-related peptide (CGRP) (**D**), neurofilament 200 (NF200) (**E**), parvalbumin (PV) (**F**), tyrosine hydroxylase (TH) (**G**), and their co-expression with mCherry-T-PSAM^4^-GlyR (Scale bars 25 um). (**H**) Percentage of each DRG neuron subpopulation that co-express mCherry-T-PSAM^4^-GlyR (n = 7 mice, (IB4: 480/973 neurons, CGRP: 435/967 neurons, NF200: 479/1014 neurons, PV: 69/219 neurons, TH: 51/261 neurons). (**I**) Example image of mCherry-T-PSAM^4^-GlyR positive afferents entering and terminating in the dorsal horn of the spinal cord (Scale Bar 100 μm, insert scale bar 25 μm). Data mean ± S.E.M.

Expression of PSAM^4^-GlyR was verified after behavioral experiments to confirm long-term expression of the construct after i.t. viral delivery. We found that our approach transduced sensory neurons in an unbiased manner (Fig. 3A). Approximately 50% of neurons in L4 DRG displayed a clear, mCherry fluorescent signal, without antibody amplification (Fig. 3B). AAV9-mCherry-T-PSAM^4^-GlyR co-expressed with specific subpopulation markers (Fig. 3C-H). Expression was comparable across different subpopulations, but with lower expression in the tyrosine hydroxylase (TH) subpopulation (Fig. 3H). We also found extensive distribution of PSAM^4^-GlyR terminals in superficial and deep layers of the spinal cord, but no spinal cord neurons were observed to express mCherry-T-PSAM^4^-GlyR (Fig. 3I). Importantly, we also found no increase of the injury marker ATF3, in DRG neurons, indicating that long-term expression of PSAM^4^-GlyR was not associated with overt neuronal damage (Fig. S7A-B). Our results suggest that i.t. viral delivery of PSAM^4^-GlyR selectively transduced different types of DRG neurons with comparable efficiency and remained stable after many months.

### PSAM^4^-GlyR mediated silencing suppressed pain-related behaviors in two inflammatory pain models

**Fig. 4:**
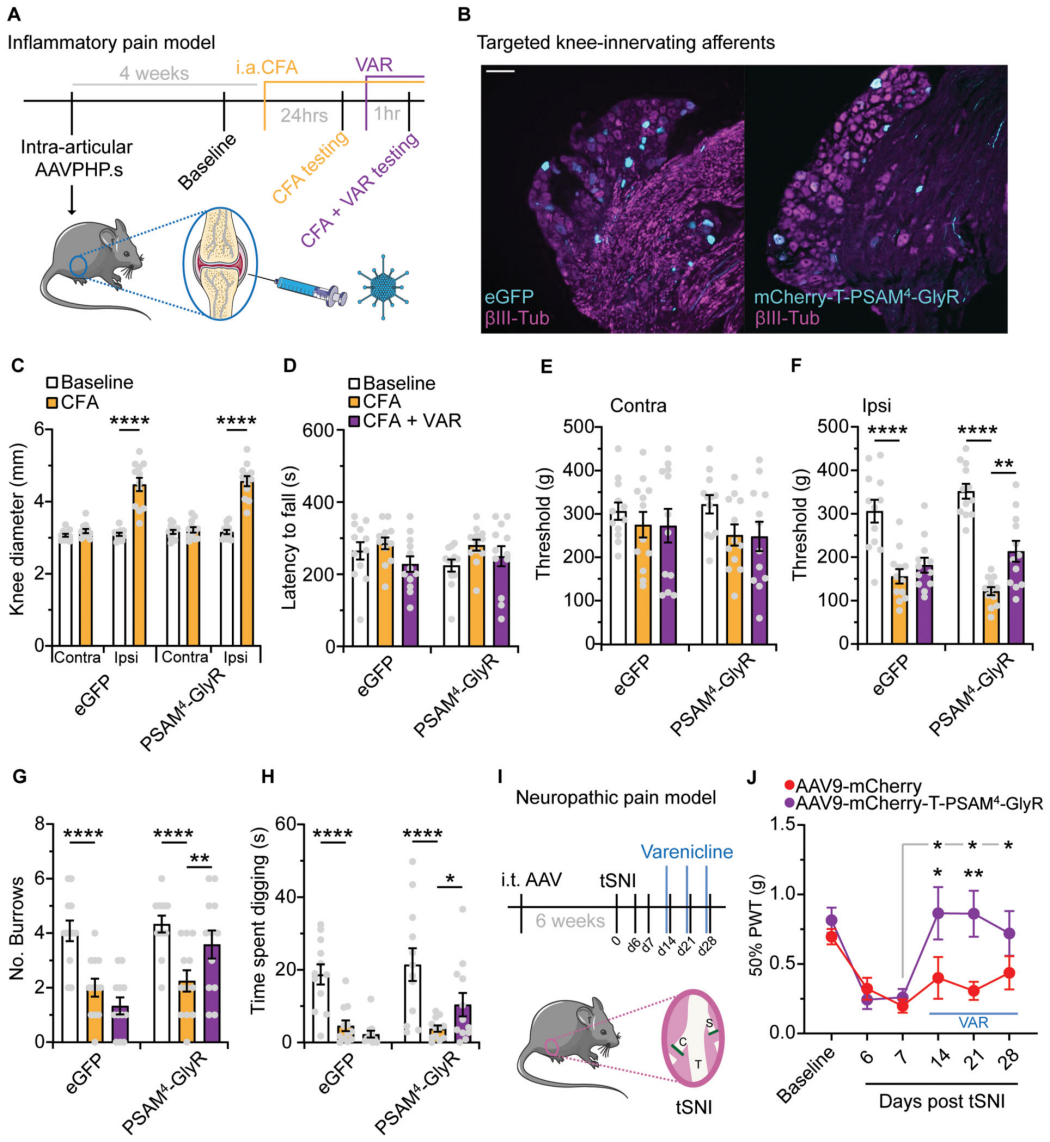
PSAM^4^-GlyR mediated silencing of inflammatory-joint and neuropathic pain. (**A**) Schematic of the experimental timeline of targeting joint afferents with AAVPHP.S, baseline testing, i.a. CFA and behavioral testing pre- and post-varenicline. (**B**) Example images of L4 DRG neurons transduced with either AAVPHP.S-eGFP or AAVPHP.S-mCherry-T-PSAM^4^-GlyR following i.a. injection (scale bar = 100 μm). (**C**) Evaluation of knee swelling following i.a. CFA (eGFP: n = 12 mice, PSAM^4^-GlyR = 12 mice, RM two-way ANOVA, post hoc Bonferroni test, **** P<0.0001). Quantification of (**D**) motor function, mechanical sensitivity of the contralateral knee (**E**), and mechanical sensitivity of the ipsilateral knee (**F**) pre CFA (baseline), post CFA and post CFA and varenicline. (**G** and **H**) Analysis of spontaneous behaviors, burrowing (**G**) and time spent digging (**H**) pre CFA (baseline), post CFA and post CFA and varenicline. eGFP: n = 12 mice, PSAM^4^-GlyR = 12 mice, RM two-way ANOVA, post hoc Bonferroni tests compared to CFA,* P < 0.05, ** P < 0.01, **** P<0.0001). (**I**) Illustration of the tibial spared-nerve injury (tSNI) neuropathic pain model and experimental timeline. Intrathecal targeting of sensory neurons, followed by tSNI and mechanical testing pre- and post-varenicline. C: common peroneal; T: tibial; S: sural. (**J**) Mechanical testing of mCherry or PSAM^4^-GlyR expressing mice before (baseline), post tSNI and post tSNI with varenicline treatment (mCherry: n = 10 mice, PSAM^4^-GlyR = 12 mice, RM two-way ANOVA, post hoc Bonferroni tests compared to D7 (grey line) or between groups, * P < 0.05, ** P < 0.01). Data expressed as mean ± S.E.M.

Following inflammation or injury it is common for nociceptors to become sensitized, showing hyperexcitability, which is key in driving pain (3). Therefore, we next asked if our humanized silencing system could be applied to pathological pain conditions. We first chose a chemical, inflammatory-like pain model where formalin was injected into the hindpaw of mice that had received an i.t. injection of either AAV9-mCherry or AAV9-mCherry-T-PSAM^4^-GlyR. All mice were dosed with varenicline 1 hr prior to the formalin assay and assessment of nocifensive behaviors were conducted. We found that mice expressing PSAM^4^-GlyR exhibited fewer pain-related behaviors in the 1^st^ phase of the formalin assay, compared to control mice (Fig. S8A-B). These results were encouraging so we undertook a more clinically relevant model of inflammatory joint pain (Fig. 4A). In this cohort of mice, we selectively targeted knee-innervating afferents using intra-articular (i.a.) AAV serotype PHP.S, as reported previously (24). After 4 weeks, mice that received either AAVPHP.S-eGFP or AAVPHP.S-mCherry-T-PSAM^4^-GlyR expressed a clear fluorescent signal in a small proportion of DRG sensory neurons (4.5 ± 0.5% in eGFP-expressing mice *vs*. 5.3 ± 0.6% in PSAM^4^-GlyR-expressing mice), with a mean cell diameter of 29.87 ± 1.0 μm (Fig. 4B). All mice then received an i.a. injection of the sensitizing agent Complete Freund’s Adjuvant (CFA). As expected, mice that received CFA developed knee swelling, ipsilateral to the injection (Fig. 4C). Mice were tested prior to CFA (Baseline), 24 hrs post CFA, and 1 hr following varenicline induced silencing. Consistent with previous findings (24), across all testing points motor function remained intact and no deficit in rotarod performance was observed (Fig. 4D). Following CFA, in both eGFP and PSAM^4^-GlyR groups, the mechanical pressure threshold of the contralateral knee was unchanged (Fig. 4E), but the ipsilateral knee became hypersensitive (Fig. 4F). Varenicline was then given to see if PSAM^4^-GlyR mediated silencing could recover inflammatory-induced mechanical hypersensitivity. Whereas no change in mechanical pressure hypersensivity occurred in the control group, a partial recovery towards baseline threshold was observed in PSAM^4^-GlyR expressing mice (Fig. 4F). We observed a similar recovery of mechanical threshold when mice were treated with the commonly used non-steroidal anti-inflammatory drug (NSAID), meloxicam (Fig. S9A-C) (39). Inflammatory joint pain can alter naturalistic-like behaviors in mice that can be used as a readout of spontaneous pain (5, 24). Following i.a. CFA all mice dug fewer burrows and spent less time digging, suggesting they were in a pain-like state (Fig. 4G-H). Following varenicline, control mice continued to exhibit reduced digging, whereas PSAM^4^-GlyR expressing mice exhibited increased digging (Fig. 4G-H). Meloxicam treatment also improved burrowing behavior after CFA (Fig. S9D-I). Collectively, our data indicate that PSAM^4^-GlyR mediated silencing of knee innervating afferents, like standard NSAID treatment, can reduce both evoked and non-evoked pain-related behaviors associated with knee inflammation.

### Activation of PSAM^4^-GlyR suppressed mechanical hypersensitivity after nerve injury

Injury, disease or genetic mutations affecting the nervous system can result in neuropathic pain. Injury to the nervous system results in sensory neuron hyperexcitability, which is thought to drive neuropathic pain (6–8). Thus, we next asked if PSAM^4^-GlyR-mediated silencing of sensory afferents can treat mechanical allodynia associated with neuropathic pain. We chose the tibial spared nerve injury model (tSNI) and measured the mechanical withdrawal thresholds of mice (40), that had previously received i.t. AAV9-mCherry or AAV9-mCherry-T-PSAM^4^-GlyR (Fig. 4I). All mice became hypersensitive to mechanical stimuli by day 7 (Fig. 4J). On days 14, 21 and 28 after tSNI, we treated all mice with the PSAM^4^-GlyR agonist varenicline, 1 hr before mechanical testing. We found that mechanical hypersensitivity recovered following varenicline treatment in mice transduced with AAV9-mCherry-T-PSAM^4^-GlyR, but not in control mice (Fig. 4J). Treatment with pregabalin, a first-line therapeutic agent for neuropathic pain also decreased mechanical hypersensitivity after tSNI (Fig. S10A), to a comparable degree to PSAM^4^-GlyR activation with varenicline (Fig. S10B-C). Therefore, PSAM^4^-GlyR activation effectively silences pain-related behavior after nerve injury.

### Activation of PSAM^4^-GlyR silences excitability in human-derived sensory neurons

**Fig. 5:**
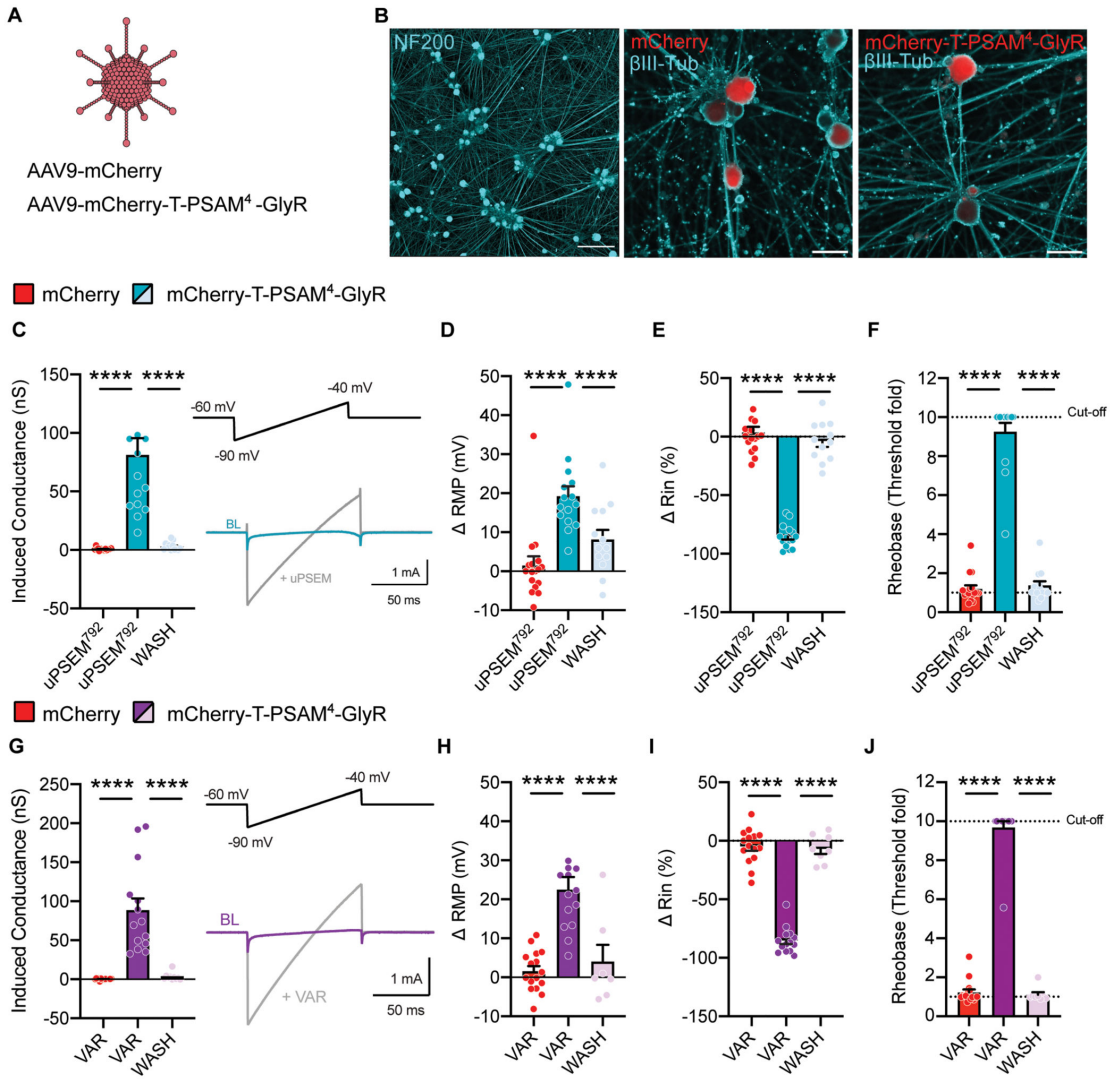
PSAM^4^-GlyR mediated silencing of human iPSC derived sensory neurons. **(A)** AAV9-mCherry or AAV9-mCherry-T-PSAM^4^-GlyR were used to virally transduce sensory neurons derived from human induced pluripotent stem cells (hiPSC-SNs). **(B)** Example images showing mature hiPSC-SNs (*left*) and mCherry labeling in AAV-mCherry (*middle*) and AAV-mCherry-T-PSAM^4^-GlyR (*right*) transduced neurons. **(C)** Quantification of membrane conductance after uPSEM^792^ application and washout. Inset shows representative traces of voltage clamp recordings used to measure membrane conductance. BL: Baseline. **(D-F)** Changes in resting membrane potential (RMP) **(B)** input resistance (Rin) **(E)** and rheobase **(F)** after uPSEM^792^ administration and washout (mCherry: n = 16 cells; mCherry-T-PSAM^4^-GlyR: n = 15 cells. One-way ANOVA with Tukey post-hoc, **** P<0.0001). **(G)** Quantification of membrane conductance after varenicline application and washout. Inset shows representative currents, used to measure membrane conductance, at baseline (BL) and post varenicline administration. **(H-J)** Changes in resting membrane potential (RMP) **(H)** input resistance (Rin) **(I)** and rheobase **(J)** after varenicline treatment and washout. (mCherry: n = 15 cells; mCherry-T-PSAM^4^-GlyR: n = 14 cells. One-way ANOVA with Tukey post-hoc, **** P<0.0001). Rheobase cut-off was defined as 10 times the baseline rheobase threshold. All data are expressed as mean ± S.E.M.

So far, we have shown the suitability of PSAM^4^-GlyR as an effective chemogenetic silencer in animal models. To demonstrate its applicability as a fully humanized system, we virally expressed PSAM^4^-GlyR in human induced pluripotent stem-cell-derived sensory neurons (hiPSC-SNs). After differentiation and maturation, we transduced hiPSC-SNs with AAV9-mCherry or AAV9-mCherry-T-PSAM^4^-GlyR (Fig. 5A). At least four weeks after viral delivery, transduced cells exhibited bright fluorescence which we used for targeted whole-cell patch clamp recordings (Fig. 5B). We found a robust increase in membrane conductance after administration of uPSEM^792^ (Fig. 5C), and thus examined the effect of activating PSAM^4^-GlyR on neuronal excitability. Application of uPSEM^792^ (10 nM) produced *V*_m_ depolarization (Fig. 5D), similar to what we observed in mouse sensory neurons. uPSEM^792^ administration also decreased input resistance (Fig. 5E), which limited the capacity of hiPSC-SNs to generate APs, as seen by an increase in rheobase (Fig. 5F). Administration of varenicline (20 nM) led to a similar increase in membrane conductance (Fig. 5G) and depolarization of the resting membrane potential (Fig. 5 H). Varenicline treatment also produced a decrease in input resistance (Fig. 5I) and an increase in rheobase, with most neurons reaching the threshold cut-off (Fig. 5J). We also measured the firing patterns of hiPSC-SNs following sustained current injections. Many cells exhibited repetitive firing in response to progressive depolarizing currents, which was abolished upon PSAM^4^-GlyR mediated silencing (Fig. S11A-B). These results suggest that PSAM^4^-GlyR activation can silence neuronal activity also in human sensory neuron models, validating its candidacy as a gene therapeutic.

### Silencing spontaneous activity in a clinical model of neuropathic pain

**Fig. 6:**
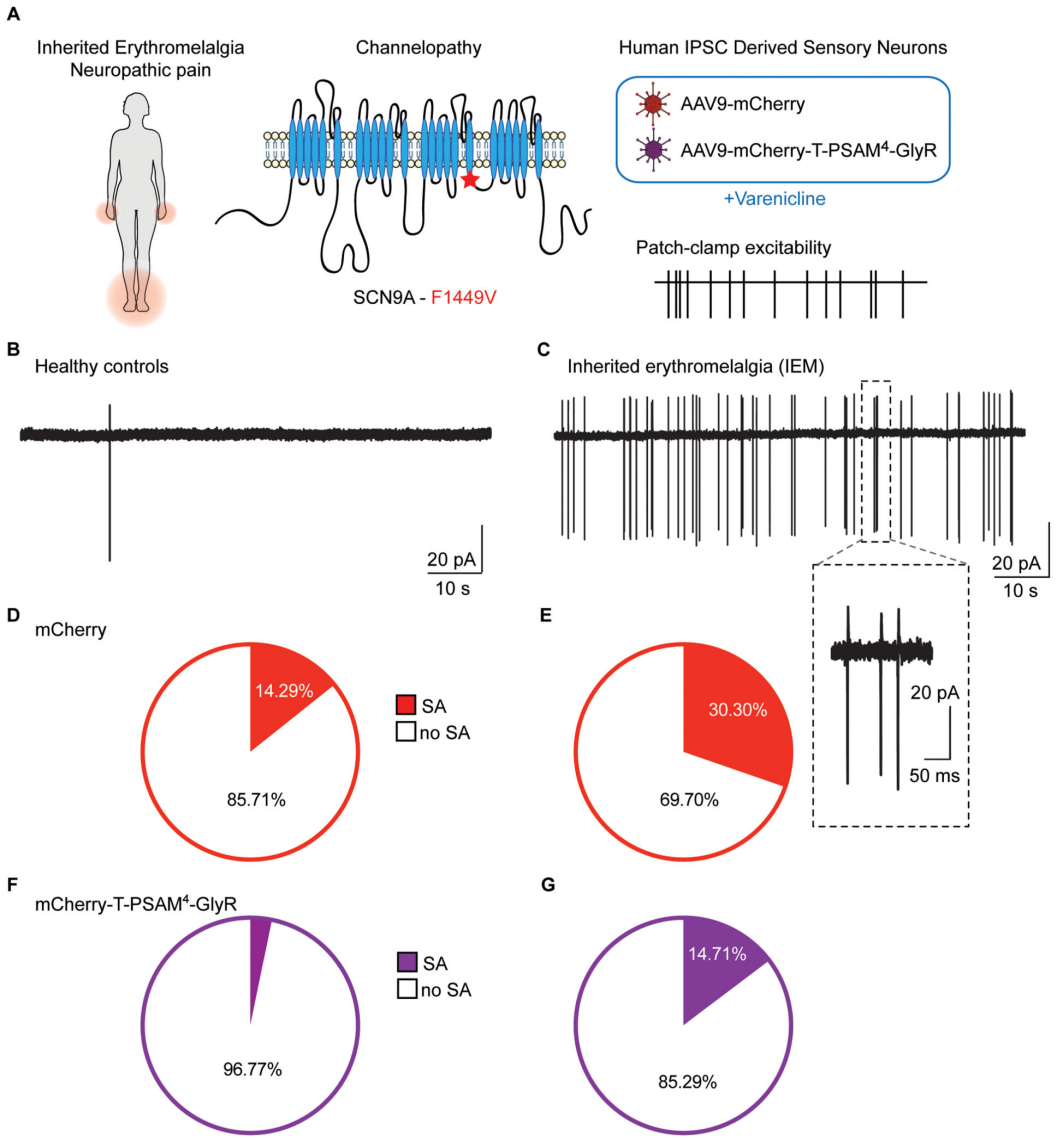
Silencing of spontaneous activity in a human neuropathic pain model. **(A)** Schematic representation of the experimental design. Patients with inherited erythromelalgia (IEM) exhibit pain and erythema in the extremities (*left*). Illustration of the point mutation (F1449V) in the Nav1.7 channel α-subunit identified in a person living with IEM (*middle*). Transduction of hiPSC-SN derived from healthy control participants and from a patient with IEM-F1449V with AAV9-mCherry and AAV9-mCherry-T-PSAM^4^-GlyR (*right*). **(B-C)** Example traces of spontaneous activity (SA) recorded in cell-attached configuration from healthy control hiPSC-SNs (**B**) and from IEM hiPSC-SNs **(C)**. Inset shows magnification of a bursting event. (**D**) Proportion of AAV9-mCherry transduced healthy control hiPSC-SNs exhibiting SA; no SA (n = 24, white) and SA (n = 4, red). **(E)** Proportion of AAV9-mCherry transduced IEM hiPSCs exhibiting SA; no SA (n = 23, white) and SA (n = 10, red). χ^2^ = 6.9, P = 0.009 **(F)** Proportion of AAV9-mCherry-T-PSAM^4^-GlyR transduced healthy control hiPSC-SNs showing SA; no SA (n = 30, white) and SA (n = 1, purple). **(G)** Proportion of AAV-mCherry-T-PSAM^4^-GlyR transduced IEM hiPSC-SNs with SA; no SA (n = 29, white) and SA (n = 5, purple). χ^2^ = 3.9, P = 0.04.

Spontaneous activity (SA) in sensory neurons is thought to be one of the key pathophysiological drivers of peripheral neuropathic pain conditions and is often well correlated with spontaneous pain experienced by patients (41, 42). Gain-of-function (GoF) mutations in *SCN9A*, the gene that encodes the voltage-gated sodium channel subunit Na_V_1.7, are associated with inherited erythromelalgia (IEM, presenting with pain and erythema of the extremities) and increased sensory neuron spontaneous activity in vivo and in vitro (43). To test the efficacy of our humanized PSAM^4^-GlyR channel in a human neuropathic pain model, we generated hiPSC-SN from iPSCs originating from a patient with IEM (GoF mutation in Na_V_1.7-F1449V), as previously reported (44) (Fig. 6A). We treated mature control and IEM hiPSC-SNs with our viruses (AAV9-mCherry or AAV9-mCherry-T-PSAM^4^-GlyR) and four weeks later measured SA in a cell-attached patch clamp configuration to avoid dialyzing the internal milieu of the cells, at 32°C (Fig. 6A). We found low frequency of spontaneous activity (SA) in cells differentiated from control patients (0.3 ± 0.2 Hz; Fig. 6B), whereas cells differentiated from the patient with IEMshowed higher SA (6.5 ± 3.7 Hz; Fig. 6C), similar to what has been previously reported (44, 45). We conducted a population-based analysis of hiPSC-SNs from healthy controls and IEM that were transduced with mCherry or mCherry-T-PSAM4-GlyR to determine whether PSAM^4^-GlyR activation by varenicline could suppress the SA observed in the IEM line. Healthy control mCherry expressing neurons exhibited a low incidence of SA (14.29%; Fig. 6D) compared to the high incidence of SA in IEM neurons (30.3%; Fig. 6E). Varenicline treatment decreased the frequency of firing activity and the proportion of spontaneously active neurons in both control (0.04 ± 0.05 Hz; 3.33%) and IEM neurons (0.004 ±0.02 Hz; 14.71%) that expressed PSAM^4^-GlyR (Fig. 6F and G). Importantly, varenicline treatment decreased the proportion of IEM neurons with SA in PSAM^4^-GlyR - expressing neurons, closer to the proportion observed in mCherry expressing control neurons (Fig. 6D). Our results highlight the translational potential of PSAM^4^-GlyR in suppressing ectopic activity in human sensory neurons, a key driver of neuropathic pain.

## Discussion

In this study, we show that expression of the human chemogenetic system PSAM^4^-GlyR in mice can silence the activity of sensory neurons and reduce behavioural hypersensitivity in inflammatory and neuropathic pain models. Viral delivery results in long-term stable expression of PSAM^4^-GlyR in different populations of sensory neurons and can be repeatedly activated in vivo. We also used PSAM^4^-GlyR to silence the excitability of human sensory neurons and to decrease ectopic discharge in patient-derived sensory neurons with a GoF Na_V_1.7 mutation causing IEM. We propose that PSAM^4^-GlyR is a promising humanised gene therapy that can be used to silence ectopic activity and hyperexcitability associated with multiple chronic pain disorders, such as inflammatory joint pain and neuropathic pain.

The inhibitory effect of opening chloride channels is highly dependent on the chloride gradient across the membrane (46). Unlike most neurons, sensory neurons maintain a high intracellular chloride concentration, that leads to chloride efflux upon chloride channel activation. However, the ensuing membrane depolarization does not produce neuronal firing of sensory neurons. Instead, the PSAM^4^-GlyR mediated chloride currents in these neurons produce strong inhibition of neuronal activity. This is likely a cumulative mechanism of two key factors. Firstly, PSAM^4^-GlyR activation drives the membrane potential toward *E*_Cl_, which would inactivate voltage-gated sodium channels (47–50). Secondly, we see a dramatic reduction in membrane resistance following PSAM^4^-GlyR activation. Together, we believe these factors render sensory neurons unable to generate and propagate APs due to chloride mediated shunting inhibition (51). Unlike sensory neurons, CNS neurons are very susceptible to the smallest changes in intracellular chloride, which affect the very nature of inhibition (hyperpolarizing/depolarizing), and may lead to the collapse of the chloride gradient, especially upon repetitive activation (52, 53). Thus, using chloride conductances as a means to suppress neuronal activity, may be particularly suited and effective in sensory neurons, compared to other neurons (32, 38). Interestingly, enhanced chloride loading in sensory neurons may also occur after inflammation and nerve injury, which depolarizes *E*_Cl_ further, leading to spiking (50), especially in combination with hyperexcitability (54). Yet, even in these pathological conditions, we believe any depolarization due to sodium ion entry can be counterbalanced by chloride flux, thus maintaining the shunting inhibition (51, 54). Indeed, we observed an increase in the degree and rate of depolarization with higher agonist concentration, but this was never associated with AP generation. Activation of PSAM^4^-GlyR effectively and reversibly silences activity in sensory neurons, and silences pain behavior even in pathological conditions such as inflammation and nerve injury.

The idea of using PSAM^4^-GlyR for therapeutic treatment of hypersensitivity in neuropathic pain conditions is of great interest. We showed that expressing the chemogenetic channel in hiPSC-SNs led to strong inhibition and complete silencing, the magnitude of which was comparable to the silencing observed in rodent neurons. Yet, this is the first report of using chemogenetics as a gene therapy to silence the activity of human sensory neurons from a cellular model of hyperexcitability and neuropathic pain. Deriving hiPSC-SNs from healthy and people living with disease is a powerful and accessible model system to test therapeutic design (55). We used this system to model inherited erythromelalgia, an inherited channelopathy, which leads to intense episodes of spontaneous, burning pain, which is often triggered by warming (43). We derived hiPSC-SNs from a patient with the F1449V GoF mutation in *SCN9A*. This mutation has been shown to result in GoF with a hyperpolarizing shift in the voltage dependence of activation of Na_V_1.7 and a depolarizing shift in steady-state inactivation resulting in lowered threshold for single APs and high frequency firing when expressed in rodent DRG neurons (56). We tested the efficacy of varenicline activation of PSAM^4^-GlyR in silencing the ectopic activity seen in hiPSC-SNs. Our data demonstrate that we can silence the aberrant activity associated with this neuropathic pain condition emphasizing the therapeutic potential of PSAM^4^-GlyR.

Previous efforts to decrease hyperexcitability in hiPSC-SNs derived from patients with IEM have focused on blocking Na_V_1.7 channels or countering their activity with dynamic clamp, both of which have produced encouraging results (44, 57). Translating these treatments to humans should provide pain amelioration without side effects, because Nav1.7 is preferentially expressed in the peripheral system, and is crucial for sensory neuron activity (43). However, targeting a single molecular target has not fared well in the clinic (19), probably because of degeneracy – changes in many ion channels and molecules may produce hyperexcitability (58) and attribution of hyper-excitability and clinical pain to a single channel mutation is rare. Our strategy of using PSAM^4^-GlyR to silence sensory neurons by-passes this problem, by efficiently suppressing ectopic activity irrespective of the underlying molecular mechanisms.

Important concerns have emerged regarding the clinical application of current chemogenetic systems. In the case of designer receptors exclusively activated by designer drugs (DREADDs), these are associated with their specific agonist CNO which is rapidly converted into neuroactive compounds that can act on other endogenous targets (28, 59), as well as producing ligand-independent changes in ion channels and second messenger signaling in sensory neurons (26). An alternative promising candidate is the engineered glutamate-gated chloride channel (GluCl) which has been shown to be effective in silencing sensory neurons (38). However, it originated from an invertebrate protein, which may raise concerns about how well it would be tolerated and integrated in humans, especially with regard to immunogenicity (38, 60). Using PSAM^4^-GlyR may circumvent these concerns, the agonist varenicline has been used safely in humans as a treatment to aid smoking cessation (61, 62). This has the advantage of already being an FDA-approved drug for human use. Furthermore, the doses required to activate the channel are in the nanomolar range, well below those used in the clinic, a feature that arises due to the mutations made in the ɑ7 nicotinic receptor part of PSAM^4^-GlyR (31). Most importantly, PSAM^4^-GlyR is engineered from human proteins, the modified human ACh nicotinic and glycine receptors. While there is always concern when expressing non-endogenous proteins, regarding long-term safety and efficacy (63, 64), PSAM^4^-GlyR would likely be better tolerated compared to proteins originating from non-mammalian systems. Importantly, we demonstrated that a single i.t. injection to deliver AAV-mCherry-T-PSAM^4^-GlyR in vivo remained stable after 11 months without any evidence of neuronal damage.

There are limitations to our present study. Therapeutic application of PSAM^4^-GlyR would require gene delivery into candidate patients. We have used i.t. and i.a. injection to deliver AAVs in animals in vivo. Viral vectors such as AAVs are widely used to deliver therapeutic genes (65). They are well tolerated in humans, and several therapeutic genes have been approved to use clinically (66, 67). However, delivering AAV-PSAM^4^-GlyR such that it effectively transduces sensory neurons in vivo in humans, remains a major challenge; i.t. injection for AAV delivery is not a trivial procedure, but has been used for gene therapy (68); i.a. injection would be similar to procedures patients undergo as part of normal therapeutic treatment for arthritis conditions, such as corticosteroid injections. Large strides are being made in the design and delivery of optimized AAV vectors with the intention of human gene therapies (69–71). Our rodent data support the use of AAVPHP.S encoding PSAM^4^-GlyR as a means of targeted delivery of our chemogenetic silencer directly to sensory neurons innervating the sensory target, such as the knee. This is of clinical interest as it is far less invasive than i.t. or intraneural injections and will result in selective silencing of sensory neurons driving the chronic pain state (69). A potential challenge of local delivery would be if injury-related changes in tissue composition and integrity could impact on sensory neuron transduction. The AAV-PSAM^4^-GlyR approach has shown broad utility across a number of diverse pain models with no observed long-term toxicity or motor dysfunction.

Further advances in focusing such a chemogenetic approach will likely arise from future targeting expression to specific sensory-neuron sub-populations. Indeed, we have good evidence that both low-threshold A-fibres, as well as C-fibre nociceptors are important in neuropathic pain (72, 73). However, we have yet to understand which sensory neuron subpopulations develop hyperexcitability and ectopic activity and contribute to specific features of inflammatory and neuropathic pain. Our viral strategy, using a generic promoter, led to broad expression in all sensory neuron subtypes, resulting in reduced pain, but also general hyposensitivity. We limited the expression of PSAM^4^-GlyR to L3-L5 DRG by i.t. injection, where input from the hindpaw is encoded and where we tested. Moving forward, generation of viral constructs dependent on Cre recombination, along with intersectional approaches, to effectively target specific sensory neuron subpopulations would be of benefit to the field in understanding pain pathophysiology (74, 75). Alternatively, AAV capsid engineering or the use of human promoters specific to sensory neuron sub-populations could also be used to focus and restrict chemogenetic receptor expression (76–78).

Taken together, our study highlights the efficacy of PSAM^4^-GlyR in silencing sensory neuron hyperexcitability and demonstrates the translational potential of an effective, stable and reversible human-based chemogenetic system for the treatment of pain.

## Materials And Methods

### Study design

The aim of our study was to demonstrate the efficacy of expressing a humanized chemogenetic chloride channel in silencing sensory neuron hyperactivity. Our objectives were to validate PSAM^4^-GlyR expression in vitro to target sensory neurons, to test the expression and efficacy of PSAM^4^-GlyR on sensory behavior in mice, and to assess the value of PSAM^4^-GlyR in control and patient derived iPSC-derived sensory neurons.

The mice used in this study were group-housed in individually ventilated cages with free access to food and water, in humidity and temperature-controlled rooms with a 12 hr light-dark cycle, in a pathogen free facility. All animal procedures adhered to the UK Home Office (Scientific Procedures) Act (1986) Amendment Regulations 2012, and were performed under a UK Home Office Project Licence. All animal experiments were carried out in accordance with University of Oxford Policy on the Use of Animals in Scientific Research. For experiments performed in Cambridge (knee joint inflammation and behavioral assessment) the University of Cambridge Animal Welfare and Ethical Review Body also approved all animal experiments. The work within this study also conforms to the ARRIVE guidelines (79). C57BL/6J wild type mice were sourced from biomedical services breeding unit at the University of Oxford and Envigo.

Sample size and experimental duration were chosen on the basis of power calculations based on our own data and previous experience in the field. Behavioral sample sizes were calculated using the software G*power2 with p-values of 0.05 and a power of >0.8. In pain behavior outcomes the effect size is taken as a 30% difference which parallels what is often seen as clinically relevant changes in pain ratings in human. Note that for group sizes in cohorts that underwent AAV intrathecal injection n of 1 was added to this sample size in order to deal with surgical attrition. If mice underwent spared nerve injury n of 1 was also added to in order to deal with any surgical attrition. All behavioral experiments were carried out by a blind experimenter on adult male and female mice. Details on animal assignment, randomization and blinding are found in specific subsections in Materials and Methods.

### Molecular cloning

pCAG-PSAM^4^-GlyR-IRES-EGFP was a gift from Scott Sternson (Addgene plasmid # 119739) and was subcloned into a pAAV vector between AAV2 ITR sites. For our purpose this construct was too large for viral production. To shorten the construct and swap the reporter, we generated a plasmid iteration that replaced EGFP with mCherry, the IRES sequence with a Tandem sequence (Furin-GSG-P2A-GSG-T2A)(80). Our initial observations noted that the PSAM4-GlyR C-terminal domain is likely sensitive to reporter tagging/linking. Therefore, we opted to swap PSAM^4^-GlyR and mCherry ORFs to generate our final construct; pAAV-CAG-mCherry-Tandem-PSAM^4^-GlyR (Addgene #208361). For detailed methods on generation of our vector see Supplementary Materials.

### Whole-cell patch clamp recordings

Voltage-clamp recordings using an Axopatch 200B amplifier and Digidata 1550 acquisition system (Molecular Devices) were performed at room temperature. Data were sampled at 20kHz and low-pass filtered at 5 kHz. Series resistance was compensated 70% –85% to reduce voltage errors. All data were analyzed by Clampfit 10 software (Molecular Devices). mCherry+ HEK cells, DRG neurons and hiPSC-sensory neurons (for culture methods see Supplementary Materials), were detected with an Olympus microscope with an inbuilt Cy3 or Texas red filter set. For recordings solutions and agonists used, see Supplementary Materials. Agonists were delivered to the cells via a perfusion system. All post agonist recordings were made 15 mins post application. Membrane conductance was measured in voltage-clamp mode with a 100ms voltage ramp from -90 mV to +40 mV every 10 s. Liquid junction potential (-13 mV) was corrected. The resultant linear current gradient was used to calculate membrane conductance using the rearranged Ohm’s law equation. Resting membrane potential (RMP) was measured in bridge mode (I=0). In current-clamp mode, neurons were held at -60 mV. Input resistance (R_in_) was derived by measuring the membrane deflection caused by a 20 pA current step. Rheobase was determined by applying 50 ms depolarizing currents of increasing steps of 25 pA until action potential (AP) generation.

For spinal cord recordings (for details on slicing procedure, see Supplementary Materials), neurons in lamina II (subtantia gelatinosa) were visually identified in an Olympus BX51 microscope equipped with infrared differential interference contrast (IR-DIC) and 40x water-immersion-objectives. Lamina II was identified by visual inspection a wide translucent band in the dorsal horn. A suction electrode filled with ACSF was placed in a dorsal root was to deliver electrical stimulation. Signals were amplified with a Axopatch 200B amplifier (Molecular Devices), digitized with a Digidata 1440 (Molecular Devices), and recorded using pClamp 10 software (Molecular Devices). Data were filtered at 5 kHz and sampled at 10 kHz. Neurons were maintained at -70 mV (corrected for liquid junction potential of -10 mV). Monosynaptic responses were determined by the absence of failures in a 10 Hz train of 10 electrical stimuli. Agonists were superfused onto slices at a rate of 2 ml/min in normal oxygenated ACSF. Only neurons with a resting potential more negative than -50 mV and stable access resistance (<25 MΩ) during the recording were included for subsequent analysis.

### Cell-attached patch clamp recordings

Cell-attached patch clamp was performed at 32°C using the same experimental set up as above and using the same solutions. Once a Giga-seal was achieved, cell-attached recordings were sampled in voltage clamp. The spontaneous activity of each cell was recorded for 5-10 mins. A spontaneously active cell was defined as firing at least 1 AP in 5 mins. Neurons were recorded in the presence of 20 nM varenicline throughout the duration of the experiment.

### Viral administration

For viral production and detailed methods, see Supplementary Materials. Briefly, neonatal pup subcutaneous injections, were carried out as follows, P5-P7 mice were briefly removed from their home cage and injected with 10 μl of virus, subcutaneously at the nape of the neck. They were rubbed in their bedding before being returned to their home cage. Mice were used for tissue or electrophysiology at least 4 weeks later. Intrathecal infusion in mice (4-6 weeks old) was performed as previously described (38), for more details see Supplementary Materials. For Intra-articular injections, AAVPHP.s-eGFP or AAVPHP.s-mCherry-T-PSAM^4^-GlyR were made to both knees of adult mice under anaesthesia. Mice then received an intra-articular injection of 10 μg complete Freund’s adjuvant (CFA; Chondrex) to induce inflammation.

### Behavioral analysis related to the hind paw

Both male and female mice were used in this study, and mice were tested at a consistent time of day, in the same environment by the same experimenter. Mice were habituated to their testing environment and equipment prior to behavioral test days. Mice we tested in a random order and randomly assigned a test box each day. The experimenter was blind to the animal group until after the behavioral analysis was complete. Mice were tested on three different days to obtain an average baseline value. Except in the case of injury, all sensory threshold data represent the average of right and left hind paws. Mice were tested after (1-2hrs unless otherwise stated) receiving i.p. uPSEM^792^ or varenicline. Mice were re-tested at least 24 hrs later for washout experiments. Mechanical, thermal, and proprioceptive tasks were carried out (See Supplementary Materials).

### Behavioural analysis related to the knee

All behavioral experiments were conducted in the presence of one female and one male investigator. Mice were allowed 30 minutes to acclimatize to the testing room before commencing behavioral assays. Both male and female mice were used in this study. Baseline behaviors were tested 2 hours prior to i.a. CFA injection. Mice were tested again 24 hrs post-CFA and 1-2 hrs post-varenicline or meloxicam. Mice were tested in a random order each time. The experimenters were blind to the animal group until after the behavioural analysis was complete. Digging ability, Motor function, and Pressure sensitivity were all carried out (see Supplementary Materials).

### Chemogenetic agonists

uPSEM^792^(30) (Tocris Bioscience) the designer PSAM^4^-GlyR agonist (31), was reconstituted in saline and injected i.p. at a final concentration of 5mg/kg (unless otherwise stated). Varenicline (Merck, PZ0004) the clinically relevant PSAM^4^-GlyR agonist, was reconstituted in saline and injected i.p. at a final concentration of 0.3mg/kg.

### Neuropathic pain model

For the spared nerve injury model we ligated and transected the sural and common peroneal branches of the sciatic nerve, leaving the tibial nerve intact (tSNI). Animals received postoperative analgesia as detailed above and were assessed behaviorally from 6 days post-SNI. Any animals that did not show a reduction in mechanical thresholds by 7 days post-SNI were excluded from further assessment and were not included in the final analysis. The lack of mechanical hypersensitivity led us to exclude 1/24 animals.

### Immunohistochemistry

Standard immunohistochemistry protocols were performed. Details of methods and antibodies used, can be found in Supplementary Materials.

### Statistical analysis

Data on variance was generated from our provisional and published data using pain outcome measures. The primary outcome was evoked behavior, experimental unit was mouse, units per group was 9 biological replicates, 3 measurements, effect size 30 (f=0.6), and RM ANOVA was chosen for the primary outcome statistical test. All data was tested for normality using the D’Agostino-Pearson normality test and the appropriate parametric or non-parametric statistical tests used accordingly. All statistical tests used were two-tailed. Statistical comparisons were made using a Student’s t-test or Mann Whitney U-test. In experimental groups in which multiple comparisons and repeated measures were made two-way analysis of variance (ANOVA) tests with appropriate post-hoc tests were performed. For electrophysiological data, n equals number of cells from at least 3 animals All data is represented as mean ± the standard error of the mean (S.E.M.) unless otherwise stated. Statistical significance is indicated as follows * P < 0.05, ** P < 0.01, *** P < 0.001, **** P <0.0001. The statistical test used is reported in the appropriate figure legend. Graph Pad prism 9 was used to perform statistical tests and graph data. Adobe illustrator CS5 were used to create schematics and medical graphics were obtained from Smart servier free medical art (smart.servier.com).

## Supplementary Material

Supplementary Materials

## Data Availability

All data and materials will be made available upon reasonable request to the corresponding author(s). Construct(s) generated in this study will be made available on Addgene.

## One Sentence Summary

Silencing of sensory neuron hyperexcitability using human-based chemogenetics shows its translational potential for pain treatment.
