# Supplementary material for: A humanized chemogenetic system inhibits murine pain-related behavior and hyperactivity in human sensory neurons
Source: Sci Transl Med. Author manuscript; Available in PMC 2023 Oct 12. (PMC7615191; doi:10.1126/scitranslmed.adh3839)
Supplement: Supplementary Materials [file EMS189055-supplement-Supplementary_Materials.pdf]

Supplementary Materials for

A humanized chemogenetic system inhibits murine pain-related behavior and hyperactivity in human sensory neurons.

Jimena Perez-Sanchez, Steven J. Middleton, Luke A. Pattison, Helen Hilton, Mosab Ali Awadelkareem, Sana R. Zuberi, Maria B. Renke, Huimin Hu, Xun Yang, Alex J. Clark, Ewan St. John Smith, David L. Bennett1

This pdf includes:

Supplementary Material and Methods

Fig. S1 to Fig. S11

Table S1 and S2

Additional Supplementary Materials

MDAR Checklist

Supplementary Materials and Methods

Vector generation

To generate our insert, (mCherry-Tandem-PSAM4-GlyR), four fragments were generated (Table S1), digested using restriction enzymes (NEB) and ligated using Quick ligase (NEB); After fragment ligation, the product (2.2kb), was PCR amplified and inserted into a pAAV-CAG backbone using NheI and AgeI restriction sites. From this construct, we next generated our pAAV-CAG-mCherry control plasmid. We PCR amplified NheI-Kozak-mCherry, and inserted it into our pAAV-CAG backbone using NheI and AgeI restriction sites. All constructs were screened and sequence confirmed using Sanger sequencing.

Viral production

pAAV-CAG-Kozak-mCherry-Tandem-PSAM4-GlyR and pAAV-CAG-Kozak-mCherry constructs were commercially packaged and serotyped with AAV9 capsid protein by the Viral Vector Facility (VVF), Neuroscience Centre Zurich (ZNZ), University of Zurich and ETH Zurich. Final titres for AAV9-CAG-mCherry-T-PSAM4-GlyR and AAV9-CAG-mCherry were 1.2 x 10^{13} vg/ml (vector genomes/milliliter) and 3.1 x 10^{13} vg/ml, respectively. pAAV-CAG-Kozak-mCherry-Tandem-PSAM4-GlyR was also commercially packaged into AAV serotype PHP.S at a final titre of 8.0 x 10^{12} vg/ml (VVF). AAVPHP.S-CAG-eGFP (v24-PHP.S) was purchased from VVF at a tire of 1.3 x 10^{13} vg/ml, and diluted to a final working tire of 8.0 x 10^{12} vg/ml (VVF).

Human embryonic kidney 293T cells and transfection

HEK293T cells were routinely cultured in Dulbecco's modified Eagle Medium (DMEM, Thermofisher scientific) and 10% foetal calf serum. Cells were periodically split using Versene solution (Gibco) and mechanical dissociation. Dissociated cells were seeded into 6 well plates and when cells reached 70% confluence, were transfected using JetPEI following the manufacturer's protocol (PolyPlus transfection). A total of 3 μg of DNA/per 35 cm well was combined with NaCl and JetPEI and after 20 mins added to HEK293T cells. The next day transfected cells were re-plated onto cover slips and used 24 hrs later.

DRG neuron culture and electroporation

Briefly, mice were sacrificed and spinal columns removed. Dorsal root ganglia were rapidly dissected and enzymatically digested at 37°C for 60-90 mins in dispase type II (4.7 mg/ml) and collagenase type II (4 mg/ml). Cells were briefly centrifuged and HBSS/CollagenaseDispase removed. Pre-warmed culture media (Neurobasal, 2% B-27 supplement, 1% Penicillin streptomyocin) was added and cells were mechanically dissociated using fire-polished pipettes. Neurons were transfected via electroporation using the Neon system (Life technologies). Dissociated cells were re-suspended in 10 μl of Buffer R plus 1 μg of total plasmid DNA per 50-100,000 cells. The electrical protocol applied was three 1500-V pulses of 10 ms duration. Cells were immediately plated on Poly-D-lysine/Laminin coated

cover slips with the addition of growth factors (mouse nerve growth factor (50 ng/ml; NGF, PeproTech) and 10 ng/ml glial-derived neurotrophic factor (GDNF, PeproTech)). Cells were used for further experiments up until day 4 *in vitro*.

Generation and culture of induced pluripotent stem cells

Healthy control iPSCs, AD2-1 and AD3-1 (StemBANCC Consortium), were derived from fibroblasts as described previously (*81*). Data obtained AD2-1 and AD3-1 hiPSCs were pooled and used as control. Another line, RCi002-A, was derived from a patient with inherited erythromelalgia and carries the F1449V mutation in SCN9A (EBiSC Consortium) (*44*). All lines were separately reprogrammed by non-integrating Sendai viral vectors using the CytoTune-iPS Reprogramming Kit (ThermoFisher). For quality control all iPSC lines were subject to strict checks before initiation of differentiation, including; tests for Sendai virus clearance, FACS for pluripotency markers, genomic integrity checks, cytoSNP analysis for copy number variation and embryoid body tri-lineage differentiation experiments. Cells are also confirmed as negative for Mycoplasma before cryopreservation. iPSCs were maintained in mTesR1 (StemCell Technologies) or StemFlex (Life Technologies) on Matrigel (Corning) coated dishes. Cells were routinely passaged at 80% confluence with EDTA (Life Technologies). Medium was supplemented with Y-27632 (Tocris) when thawing iPSCs.

Differentiation of human induced pluripotent stem cells to sensory neurons

Human iPSCs were differentiated following the Chamber's protocol, with modifications (*81, 82*). In brief, cells were passaged using Versene EDTA (ThermoFisher) and plated at high density. Neural induction commenced with the addition of SMAD inhibitors SB431542 (Sigma, 10 mM) and LDN-193189 (Sigma, 100 nM) to KSR base medium (Knockout-DMEM, 15% knockout-serum replacement, 1% Glutamax, 1% nonessential amino acids, 100 mM b-mercaptoethanol, (ThermoFisher)). Three additional small molecules were introduced on day 3 (CHIR99021 (Sigma, 3 mM), SU5402 (Sigma, 10 mM) and DAPT (Sigma, 10 mM). The dual SMAD inhibitors were withdrawn on day 5. The base medium was gradually transitioned to N2/B27 medium (Neurobasal medium, 2% B27 supplement, 1% N2 supplement, 1% Glutamax, (ThermoFisher)) in 25% increments. Cells were replated onto glass coverslips at day 12 of the differentiation in N2/B27 medium supplemented with four recombinant growth factors at 25ng/ml (BDNF; ThermoFisher, NT3, NGF, GDNF; Peprotech). CHIR90221 was included for 4 further days. Medium changes were performed twice weekly after replating onto coverslips. If required, Cytosine b-D-arabinofuranoside (araC, 1-2 mM, Sigma) was included in the medium soon after replating to kill the few non-neuronal dividing cells remaining in the culture. AraC was withdrawn from the medium once a pure neuronal culture was obtained, as judged by the absence of morphologically non-neuronal cells on phase-contrast light microscopy. This state was typically achieved 2-3 weeks after replating. From day 28, the concentration of all four recombinant growth factors was reduced to 10ng/ml. Phenol-free Matrigel (Corning, 1:500 dilution) was included in all medium changes from day 28 onward. Medium changes were performed twice weekly. AAVs (AAV9-CAG-mCherry-T-PSAM^4-GlyR, multiplicity of infection (MOI): 1M, and AAV9-CAG-mCherry, MOI: 100K) were added to the cultures around day 50. AAVs remained in culture for 7 days without media change. Biweekly media changes resumed thereafter. Cells were used for experiments at least 4-6 weeks post AAV infection.

Whole-cell patch clamp recording solutions

Borosilicate glass capillaries (1.5 mm OD, 0.84 mm ID; World Precision Instruments) were pulled on a horizontal puller (P-1000; Sutter Instruments) to form patch pipettes of 2–5 MΩ tip resistance and filled with an internal solution containing (mM): 100 K-gluconate, 28 KCl, 1 $MgCl_2$, 5 MgATP, 10 HEPES, and 0.5 EGTA; pH was adjusted to 7.3 with KOH and osmolarity set at 305-310 mOsm (using glucose). Cells were maintained in a chamber constantly perfused with a physiological extracellular buffer containing (mM): 140 NaCl, 4.7 KCl, 2.5 $CaCl_2$, 1.2 $MgCl_2$, 10 HEPES and 10 glucose; pH was adjusted to 7.4 with NaOH and osmolarity set at 310-315 mOsm (using glucose). For slices, patch pipettes (5–7 MΩ) were pulled on a horizontal puller (P-1000; Sutter) and filled with the following intracellular solution (in mM): 135 K-gluconate, 5 KCl, 2 $MgCl_2$, 10 HEPES, 4 ATP-Na, 0.4 GTP-Na, 0.1% Lucifer-Yellow (LY, Sigma), pH 7.3 adjusted with KOH. $uPSEM^{792}$ and varenicline fresh daily, diluted in extracellular buffer to a final concentration of 10 nM and 20 nM respectively, unless otherwise stated.

Spinal cord slice preparation

Adult mice, that had received a subcutaneous viral injection when neonates, were anaesthetised with ketamine/xylazine (i.p. 90mg/kg / 10mg/kg), and perfused transcardially with ice-cold oxygenated (95% O_2, 5% CO_2) sucrose-based artificial cerebrospinal fluid (sACSF) containing (in mM): 100 sucrose, 63 NaCl, 2.5 KCl, 1.2 $MgCl_2$, 1.2 NaH_2PO_4, 25 $NaHCO_3$, 25 glucose and 1 kynurenate. Spinal cords were carefully obtained by laminectomy in ice-cold sACSF with dorsal roots attached. Parasagittal slices (300 μm) were cut on a vibratome (Leica VT 1200) in ice-cold sACSF. Slices were then transferred to a submerged chamber containing oxygenated NMDG-based recovery ACSF (rACSF) for 15 minutes at 34 °C, containing (in mM): 93 NMDG, 2.5 KCl, 1.2 NaH_2PO_4, 30 $NaHCO_3$, 20 HEPES, 25 Glucose, 5 Na ascorbate, 2 thiourea, 3 Na pyruvate, 10 $MgSO_4$ and 0.5 $CaCl_2$, and adjusted to pH 7.4 with HCl. After recovery incubation, slices were transferred to oxygenated ACSF where they were maintained at room temperature prior to transfer to the recording chamber. ACSF was composed of (in mM): 126 NaCl, 2.5 KCl, 2 $MgCl_2$, 2 $CaCl_2$, 1.25 NaH_2PO_4, 26 $NaHCO_3$ and 10 glucose.

Intrathecal infusion

Each animal was anaesthetised using 2% isoflurane and prepared for surgery by shaving a region over the thoracic vertebrae. T-10 and T-11 vertebrae were located, an incision was made followed by removal of soft tissue to expose the dura and spinal cord. A drop of lidocaine was applied to the dura for approximately 1-2 mins then removed. Using a 30 gauge needle the dura was carefully punctured (CSF leak at this point suggested a successful puncture). A cannula system was designed by connecting tubing of decreasing size until the final cannula tip measured 0.008 in (O.D) x 0.004 in (I.D). The end of the cannula was inserted approximately 1 cm caudal into the subdural space. Using a syringe pump driver, 8 μl of AAV was injected into the subdural space at a rate of 1 μl/min. Following injection, the cannula was allowed to rest in position for 2 min before being slowly removed. The dura was coated with a single drop of dura gel (Cambridge NeuroCare) to seal the dura and prevent further CSF leak. Finally, the incision site was sutured closed

and appropriate post-operative care and analgesics given (local 2 mg/kg Marcain, AstraZeneca and systemic 5 mg/kg Rimadyl, Pfizer). Animals were used for behaviour or histology at least 6 weeks post-surgery.

Intra-articular injection

Intra-articular injections of AAVPHP.s-eGFP or AAVPHP.s-mCherry-T-PSAM^4-GlyR were made to both knees under anaesthesia (100 mg/kg ketamine and 10 mg/kg xylazine, delivered intraperitoneally) when mice were aged 6 weeks. 4-weeks later, after capturing baseline behaviours, mice were anaesthetised and one knee (side determined randomly) received an intra-articular injection of 10 µg complete Freund's adjuvant (CFA; Chondrex) to induce inflammation. The width of each knee joint was measured with digital callipers before and 24-hours post-CFA injection. After assessing post-CFA behaviour, mice received an intraperitoneal injection of 0.3 mg/kg varenicline (from a 0.06 mg/ml stock).

Analgesic drugs and recovery scores

Pregabalin (ORB389663-BOR, Stratech) was used as a positive control for tSNI experiments. 10 mg/kg was given 1hr prior to von Frey testing on day 14 post SNI. Meloxicam (Metacam, 5 mg/kg; Boehringer Ingelheim) was used as a positive control for knee inflammation experiments. Meloxicam, or saline, mice were tested in a random order each time. Recovery achieved was calculated using the following (Drug data-injury data)/(Baseline data-Injury data), expressed as a percentage. Less than 0 = behavior was worse, 0 = no recovery, 100 = full recovery, more than 100 exceeds full recovery (which would reflect mice being less sensitive that their original baseline).

Behavioural Assays

von Frey

Mice were elevated on a wire mesh base in a test box (5 × 5 × 10 cm), and acclimatised to the equipment for 30–60 min. The plantar hind paws were tested using calibrated von Frey hairs (Linton Instrumentation) using the 'up-down' method (Dixson 1980) to evaluate their 50% paw withdrawal thresholds. For spared nerve injury experiments mice were test for mechanical sensitivity over the course of the injury (days 6, 7, 14, 21, 28).

Brush

The plantar hind paws of mice were brushed (1 cm s-1) with a fine artists paint brush. Each mouse received 5 successive stimuli on alternate hind paws (10s apart), twice. The number of responses were recorded. A response included, lifting, flicking or moving the hind paw or walking away from the stimulus.

Pinprick

Noxious mechanosensation was assessed by the pinprick test described previously (*83*) Mice were housed and acclimatised similarly to the von Frey test. Mice were tested on their plantar hind paws using a sharp pin attached to a 1 g calibrated von Frey filament. Mice were video recorded using a GoPro at 240 fps, and the latency to withdraw from the pinprick analysed by an investigator blind to treatment groups. Three measurements were taken for each hind paw per trial and plotted latency represents the average of both paws.

Hargreaves

Thermal thresholds were assessed using an infrared light source applied to the plantar surface of each hind paw. Three measurements were taken for each hind paw and the averaged latency to withdraw was measured.

Dry Ice

Noxious cold thresholds were measured using the dry ice assay. Mice were elevated on a borosilicate glass (5 mm) platform in a test box. Pieces of dry ice were place into a 2 ml syringe (top cut off). The syringe filled with dry ice was placed against the glass from below (where hind paws were flat and visible). Latency to withdraw paws form the dry ice/glass was measured. Three measurements were taken for each hind paw

Beam task

The Beam test (*84*) apparatus consisted of a 1-m long horizontal beam of 12-mm width suspended from the ground. Mice were placed at one end of the beam next to a light source and walked across the beam towards a darkened box/house. Trials were recorded once per day and video footage was used by an investigator blind to treatment groups, to assess number of steps and number of missteps. Baseline data were generated from the average of two trials on separate days. 1 mouse was excluded from the beam test due to abdominal obesity, which prevented the hind paws from being able to reach the beam.

Digging

The digging behaviour of mice was measured as a readout of spontaneous pain. Testing involved placing individual mice into standard 49 × 10 × 12 cm cages filled with ~4 cm tightly packed fine-grain aspen midi wood chip bedding substrate (LBS Biotechnology). Mice were allowed 3 minutes to explore testing cages under video surveillance. Training sessions were carried out the day before baseline behaviours were captured, during these sessions mice were placed in test cages as per a normal test, however, mice that did not dig for at least 15 seconds were subsequently placed in a test cage with a cage-mate that did meet this criterion until both animals demonstrated digging behaviour. Following test digs, the number of visible burrows at the end of the 3 minutes was recorded. Digging duration was scored independently by two investigators following the conclusion of each study and blinding of the acquired videos; since the scores of investigators was well correlated (R2 = 0.84, across 174 videos) an average is reported as the digging duration.

Rotarod

Locomotor function and coordination was assessed using a rotarod (Ugo Basile). Mice were placed on the rotarod at a constant speed (7 rpm) for 1 minute before starting an accelerating programme (7-40 rpm, over 5 minutes), test runs were video recorded. Mice were removed from the rotarod if they fell, following two consecutive passive rotations or after 6 minutes of the accelerating program, whichever occurred first. Mice were first placed on the rotarod the day before baseline behaviours were captured to gain some familiarity with the assay. The latency to passive rotation or fall was timed by 1 investigator at the conclusion of each study following blinding of the acquired videos.

Pressure Application Measurement

Mechanical sensitivity of the knee joint was assessed using a pressure application measurement device (Ugo Basile). Mice received no training in this assay before acquiring baseline sensitivity and digging, and rotarod behaviours were always assessed before the application of pressure to the knee joint. Animals were scruffed before the force transducer

was used to apply gradual force to each of the animals' knee joints, by squeezing the joint medially. The withdrawal threshold was recorded when an animal withdrew the limb being tested, or after 450 g force was applied, whichever occurred first. Each animal was tested twice per time point, with a short break between tests, withdrawal force is reported as an average of the two measurements taken at each time point.

Chemical pain model - formalin assay

Mice were chosen at random from their home cage and the left hind paw of each mouse was injected subcutaneously with 2% formalin and mice were immediately placed in a test box (5 x 5 x 10 cm), on a glass base, which was elevated above a camera. The perimeter and roof of the test box consisted of mirrors to allow good visualisation of the injected hindpaw. The mice were video recorded in this environment for 1 hr while the experimenter left the room. Off line analysis was used to measure nocifensive behaviours of the injected hind paw (lifting, licking, flinching, shaking) every 5 mins for 60 mins. The formalin assay was also analysed in 2 phases; 1st phase 0-15 mins, 2nd phase 15-60 mins. All formalin behaviour was conducted 1 hrs post 0.3mg/kg i.p. of varenicline.

Immunohistochemistry

Animals were deeply anaesthetised with pentobarbital and the blood cleared from all tissues by perfusing saline through the vascular system. Mice were then perfuse-fixed using 4% paraformaldehyde (PFA). Tissues were then collected and post-fixed in 4% PFA accordingly (DRG: 1–2 h, spinal cord: 24 h). All tissues were cryoprotected in 30% sucrose for a minimum of 48 h, followed by embedding the tissue and sectioning on a cryostat. (DRG: 12 μm, spinal cord: 15 μm). Cultured cells were fixed with 4% PFA for 10 min and treated similarly to other tissues. Standard immunohistochemistry protocols were used. Briefly, fixed/sectioned samples were washed in PBS and blocked in a blocking solution (5% normal donkey serum, 0.3% TritonX-100, PBS) for 1hr at room temperature (RT). Primary antibodies (Table S1) were diluted in blocking solution and applied to tissue or cells overnight at RT. The next day samples were washed in a wash solution (0.3% TritonX100, PBS) followed by a 2hr incubation with secondary antibodies diluted in wash solution at RT. Samples were mounted using Vectorshield and imaged on a confocal microscope (Zeiss LSM-710). Images were analysed using Fuji/ImageJ (NIH). For quantification at least three sections per animal were used, with at least 3 animals per group.

Supplementary Figures:

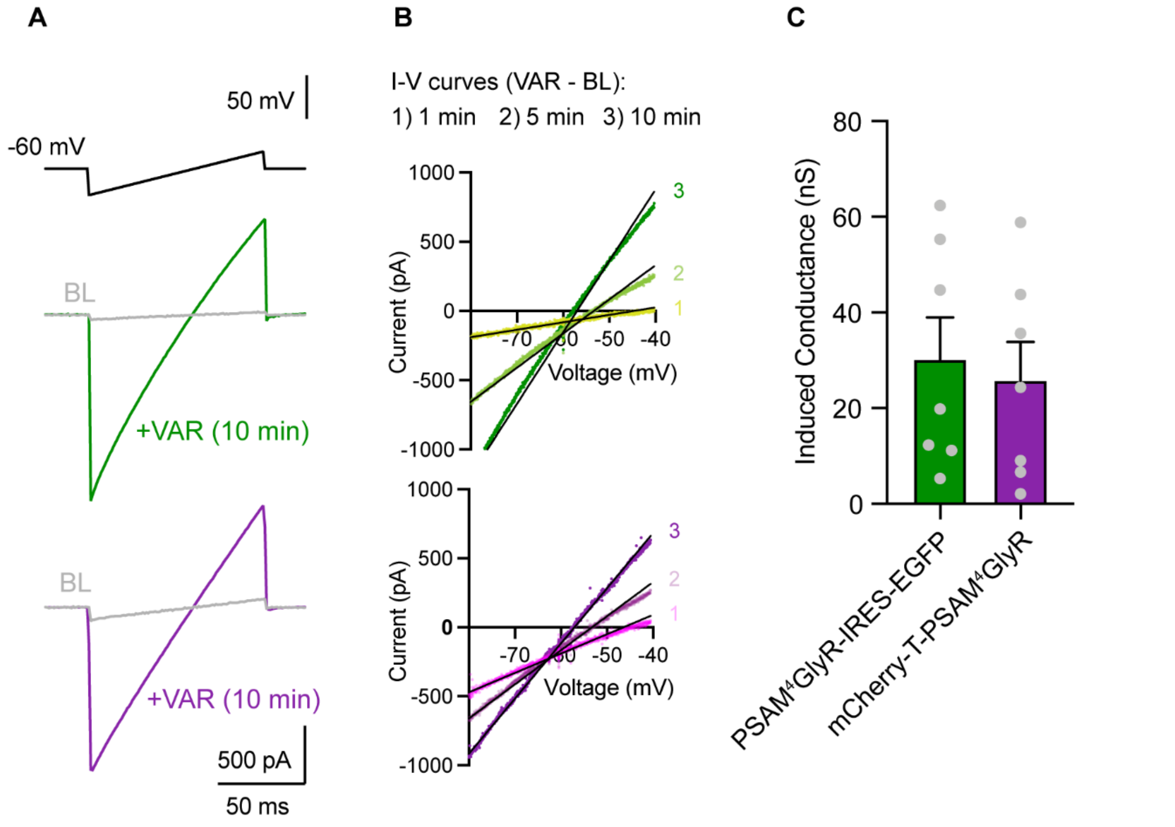

Fig. S1. Modification of the construct containing PSAM^4-GlyR does not affect function.

(A) Representative traces showing increased membrane conductance after varenicline administration in HEK293t cells transduced with PSAM^4-GlyR-IRES-GFP (green) and mCherry-T-PSAM^4-GlyR (purple). Holding current (V_h) was -60 mV. BL: Baseline; VAR: varenicline. **(B)** I-V curves from the cells in **A** obtained at different time points from the administration of varenicline (1, 5 and 10 mins). Baseline current was subtracted from the currents after agonist administration (VAR-BL). A linear regression was fitted for each time point to estimate the current reversal potential (E_{rev}). At the beginning of varenicline treatment, E_{rev} was -44 mV in PSAM^4-GlyR-IRES-GFP cells and -46 mV close to the estimated E_{Cl}. As the channel remained opened, E_{rev} progressively approached V_h. (**C**) Quantification of induced membrane conductance in HEK293t cells after varenicline administration (PSAM^4-GlyR-IRES-GFP n = 7 cells, mCherry-T-PSAM^4-GlyR n = 7 cells, Unpaired t-test, P > 0.05). Data expressed as mean ± S.E.M.

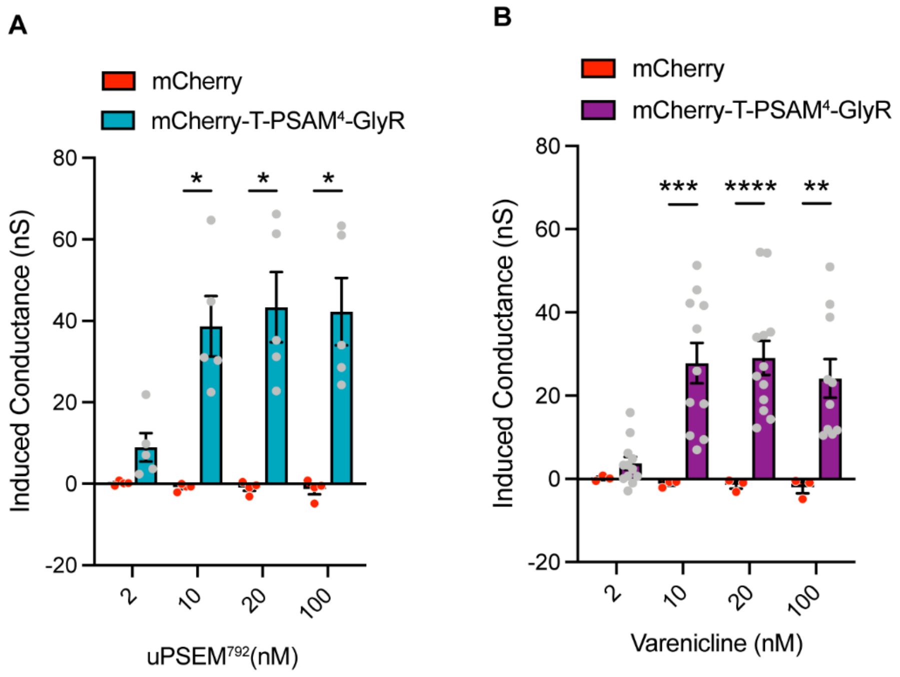

Fig. S2. Dosage of PSAM^4-GlyR agonist required for increase in membrane conductance.

(A) Quantification of changes in membrane conductance by application of 2, 10, 20 and 100 nM uPSEM^{792} in dissociated sensory neurons transduced with mCherry (n = 4) or mCherry-T-PSAM^4-GlyR (n = 5; RM-two way ANOVA, post-hoc Bonferroni test, ** P = 0.001, *** P = 0.0005, **** P < 0.0001). **(B)** Quantification of membrane conductance after application of 2, 10, 20 and 100 nM varenicline in dissociated sensory neurons transduced with mCherry (n = 3) or mCherry-T-PSAM^4-GlyR (n = 11; RM-two way ANOVA, post-hoc Bonferroni test, * P < 0.05). Data expressed as mean ± S.E.M.

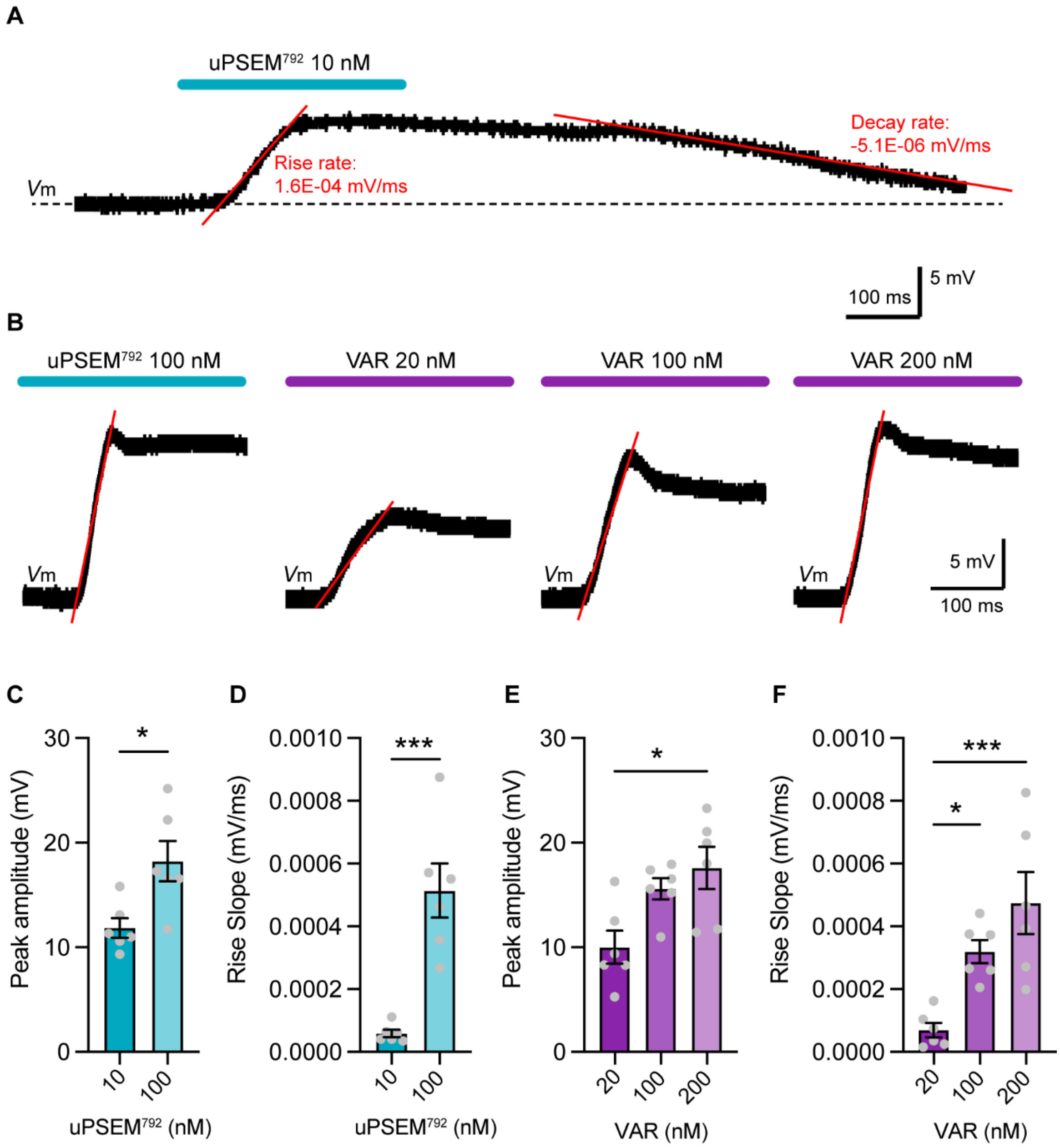

Fig. S3. Dynamics of membrane potential depolarization following PSAM[4]-GlyR activation in sensory neurons. **(A)** Representative trace of membrane potential (V_m) after application of 10 nM uPSEM[792] to sensory neurons expressing mCherry-T-PSAM[4]-GlyR. The red lines show the rate of depolarization (Rise slope) and repolarization (Decay slope). **(B)** The rate of depolarization to a higher concentration of uPSEM[792] (100 nM) and to different doses of varenicline (VAR). **(C-D)** Quantification of peak amplitude **(C)** and rise slope **(D)** of V_m with uPSEM[792] administration at the dose used in the study (n = 6) and 10-fold higher (n = 6). Unpaired t-test; * P < 0.05, *** P < 0.001. **(E-F)** Quantification of peak amplitude **(E)** and rise slope **(F)** of V_m obtained with the dose of varenicline used in this study (20 nM; n=6) and 5- and 10-fold higher concentrations (n = 6). One-way ANOVA, post-hoc Tukey test; * P < 0.05, *** P < 0.001. Data expressed as mean ± S.E.M.

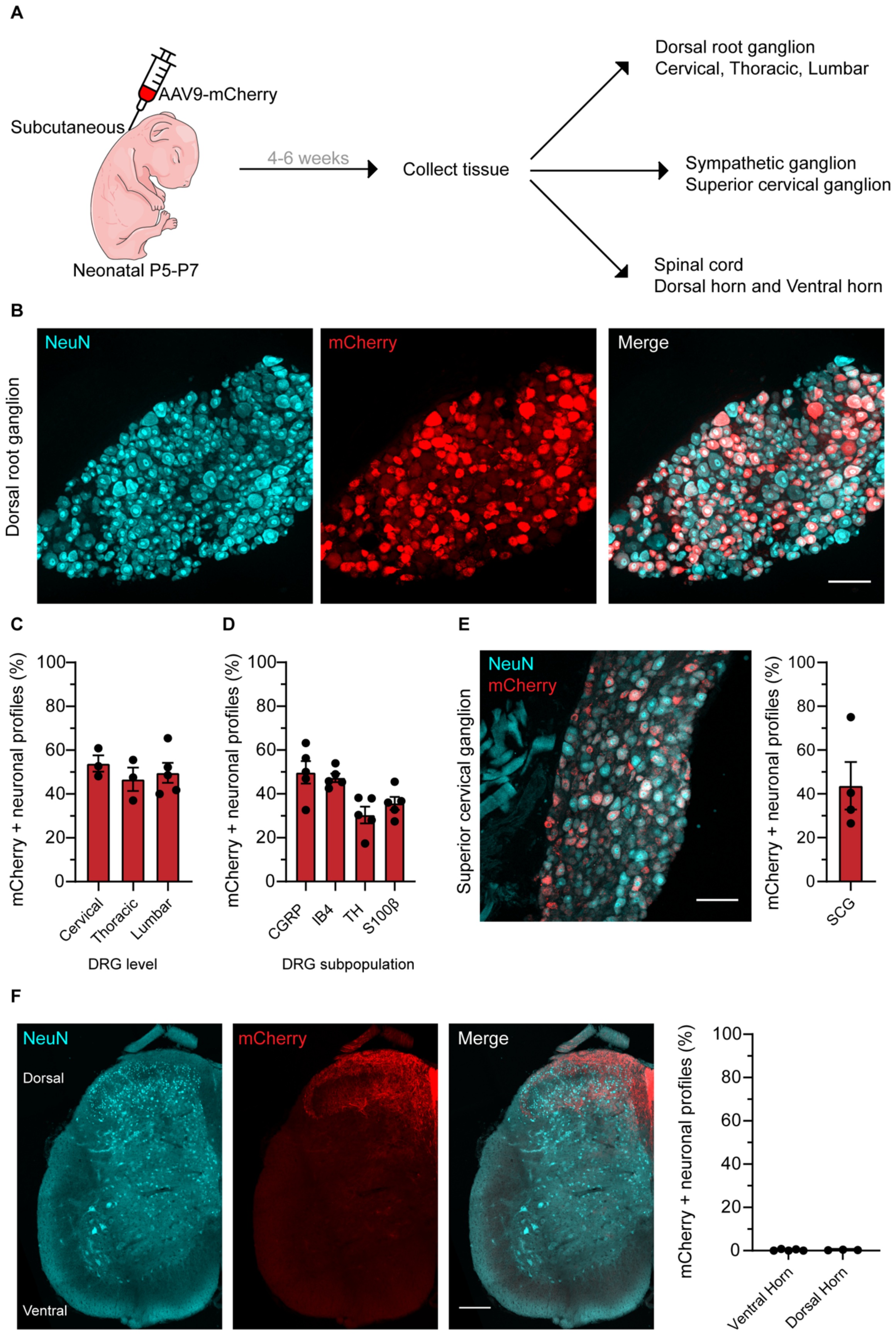
A
AAV9-mCherry
Subcutaneous
Neonatal P5-P7
4-6 weeks
Collect tissue
Dorsal root ganglion
Cervical, Thoracic, Lumbar
Sympathetic ganglion
Superior cervical ganglion
Spinal cord
Dorsal horn and Ventral horn
B
Dorsal root ganglion
NeuN
mCherry
Merge
C
mCherry + neuronal profiles (%)
Cervical
Thoracic
Lumbar
DRG level
D
mCherry + neuronal profiles (%)
CGRP
IB4
TH
S100β
DRG subpopulation
E
Superior cervical ganglion
NeuN
mCherry
mCherry + neuronal profiles (%)
SCG
F
NeuN
Dorsal
Ventral
mCherry
Merge
mCherry + neuronal profiles (%)
Ventral Horn
Dorsal Horn

Fig. S4. Subcutaneous injection of AAV9 in neonatal mice, specifically targets peripheral ganglia, and not spinal neurons.

(A) Illustration of experimental and analytical design. Neonatal mice, postnatal days 5-7, received one 10ul subcutaneous injection of AAV9-mCherry at the nape of the neck. 4-6 week later tissues (dorsal root ganglia, sympathetic ganglia and spinal cords) were collected and analysed for mCherry expression. **(B)** Example image of L4 DRG neurons transduced with AAV9-mCherry. Scale bar 100 µm. **(C)** Quantification of DRG neurons transduced with AAV9-mCherry across cervical, thoracic and lumbar levels. **(D)** AAV9-mCherry transduced the four major DRG subpopulations. **(E)** The superior cervical ganglion (sympathetic ganglion) was also transduced by AAV9-mCherry. Scale bar 100 µm. **(F)** mCherry positive primary afferent terminals can be visualised in the dorsal horn of the spinal cord, following neonatal s.c. injection of AAV9-mCherry. mCherry positive spinal cord neurons were not identified. Scale bar 200 µm. Data mean ± S.E.M.

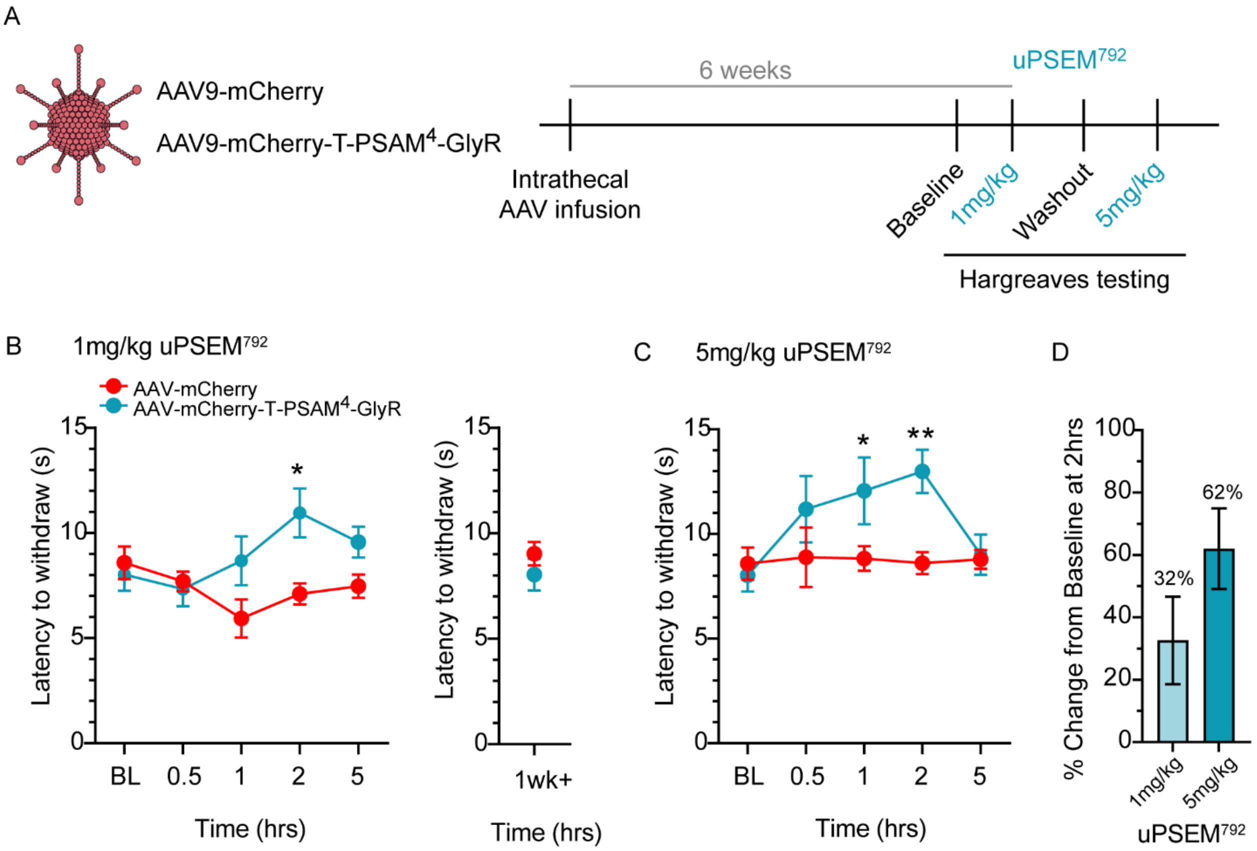

Fig. S5. uPSEM^{792} dosage and time-course of agonist induced PSAM^{4}-GlyR silencing of thermal nociception. (**A**) Schematic of the experimental timeline, AAVs were delivered intrathecally and 6 weeks later Hargreaves testing was conducted post i.p. of 1mg/kg or 5mg/kg uPSEM^{792}. (**B**) The latency to withdraw from a noxious radiant heat source was measured 0.5, 1, 2, and 5 hrs post 1mg/kg uPSEM^{792} (mCherry: n = 6 mice, PSAM^{4}-GlyR: n = 8 mice, RM- two way ANOVA, post-hoc Bonferroni test, * P = 0.013). Behavioral hyposensitivity returned to normal after 5 hrs and remained normal for 1 week. (**C**) The latency of AAV9-mCherry-T-PSAM^{4}-GlyR mice to withdraw from a radiant heat source, post 5mg/kg uPSEM^{792} compared to AAV9-mCherry mice (mCherry: n = 3 mice, PSAM^{4}-GlyR: n = 4 mice, RM- two way ANOVA, post-hoc Bonferroni test, * P < 0.05, ** P < 0.01). (**D**) The percentage change in withdrawal latency at 2hrs (from baseline) for 1mg/kg (n = 8 mice) and 5mg/kg uPSEM^{792} (n = 4 mice). Data expressed as mean ± S.E.M.

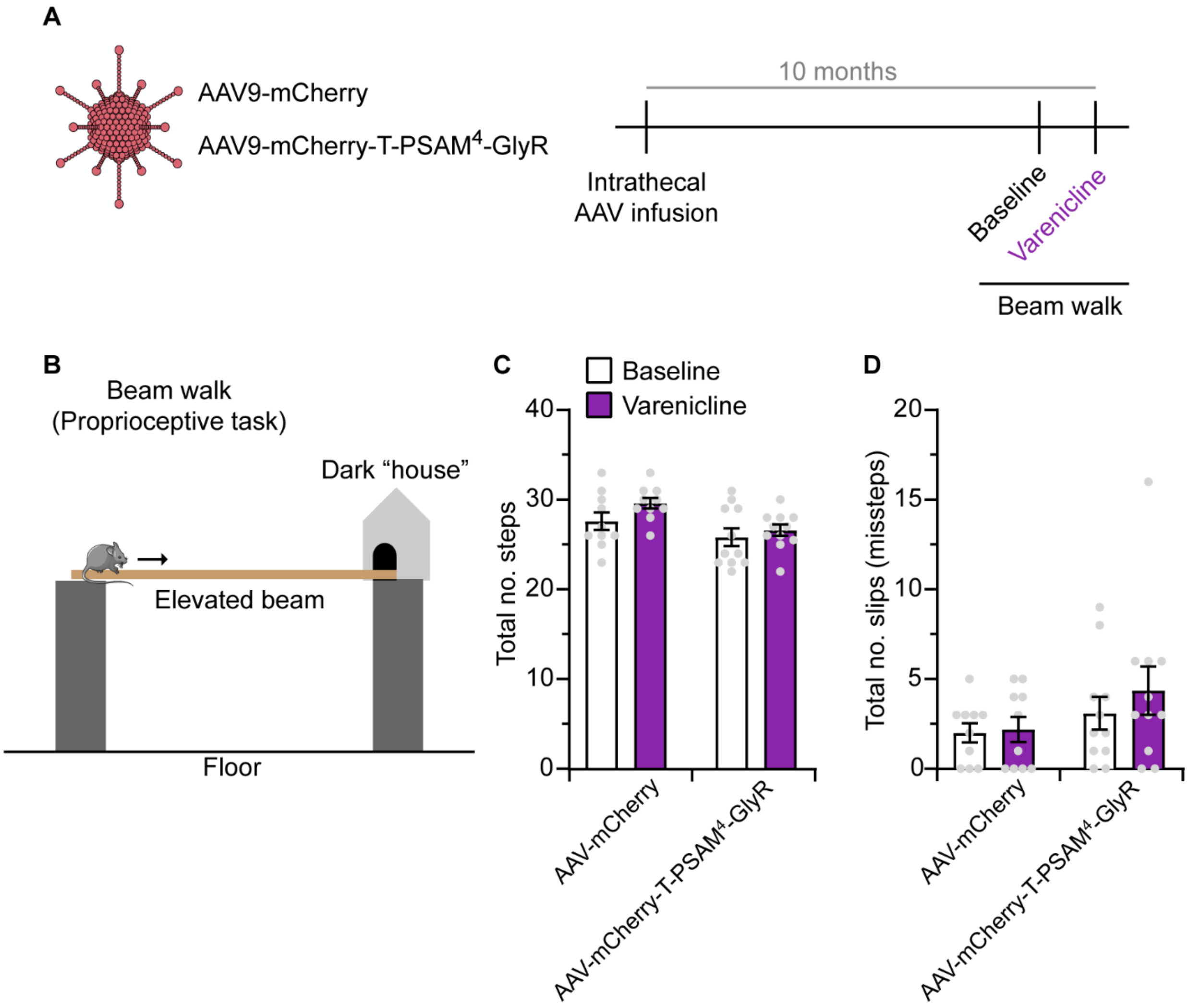

Fig. S6. Proprioceptive behaviors are preserved following PSAM^4-GlyR mediated silencing of sensory neurons. (**A**) Schematic of the experimental design. (**B**) Depiction of the beam task, mice were trained to walk along a thin beam toward a dark house/space. Steps along the beam were video recorded. (**C**) Quantification of total number of steps during the beam task. (**D**) Quantification of the total number of slips or mistakes during the task. (mCherry: n = 10 mice, PSAM^4-GlyR: n = 11 mice, all data sets RM-two way ANOVA, post-hoc Bonferroni test, P > 0.05). Data expressed as mean ± S.E.M.

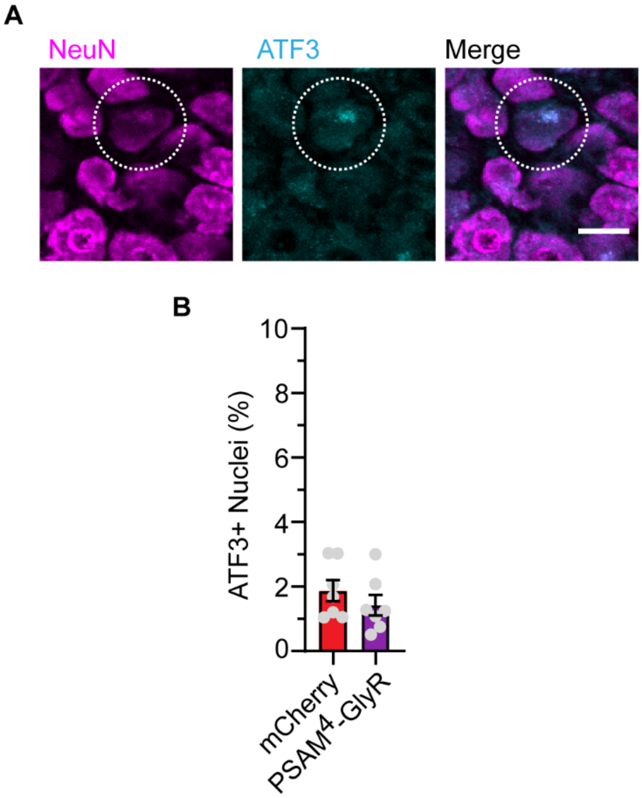

Fig. S7. Long-term expression of mCherry-T-PSAM^4-GlyR, does not result in up regulation of the injury marker ATF3.

(**A**) Example image of a DRG neuron with an ATF3 positive nucleus (scale bar 25 um). (**B**) Quantification of the percentage of ATF3 + nuclei in DRG neurons from mice that received AAV9-mCherry or AAV9-mCherry-T-PSAM^4-GlyR (mCherry: n = 7 mice, 19/1014 neurons, PSAM^4-GlyR: n = 7 mice, 16/1324 neurons). Data expressed as mean ± S.E.M. n.s. $P > 0.05$, calculated by an unpaired t-test.

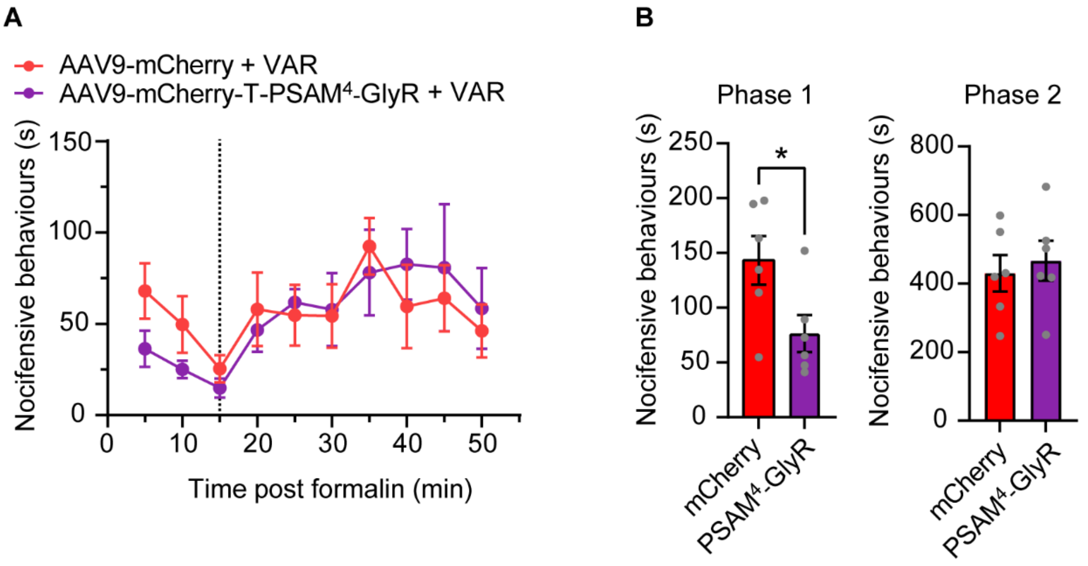

Fig. S8. PSAM^4-GlyR chemogenetic silencing of primary afferents reduces chemical-induced pain.

(**A**) AAV9-mCherry or AAV9-mCherry-T-PSAM^4-GlyR mice were both given varenicline and 1 hr later received an injection of 2% formalin in the hind paw. Nocifensive behaviours over the following 50 mins were measured. The vertical line at 15 minutes represents the transition from phase 1 to phase 2 of the formalin test (**B**) Quantification of nocifensive behavior duration in phase 1 (0-15 mins) and phase 2 (15-50 mins) of the formalin test, of mCherry or PSAM^4-GlyR expressing mice, following varenicline (mCherry: n = 6 mice, PSAM^4-GlyR: n = 6 mice, unpaired t-test, * $P < 0.05$). Data expressed as mean ± S.E.M.

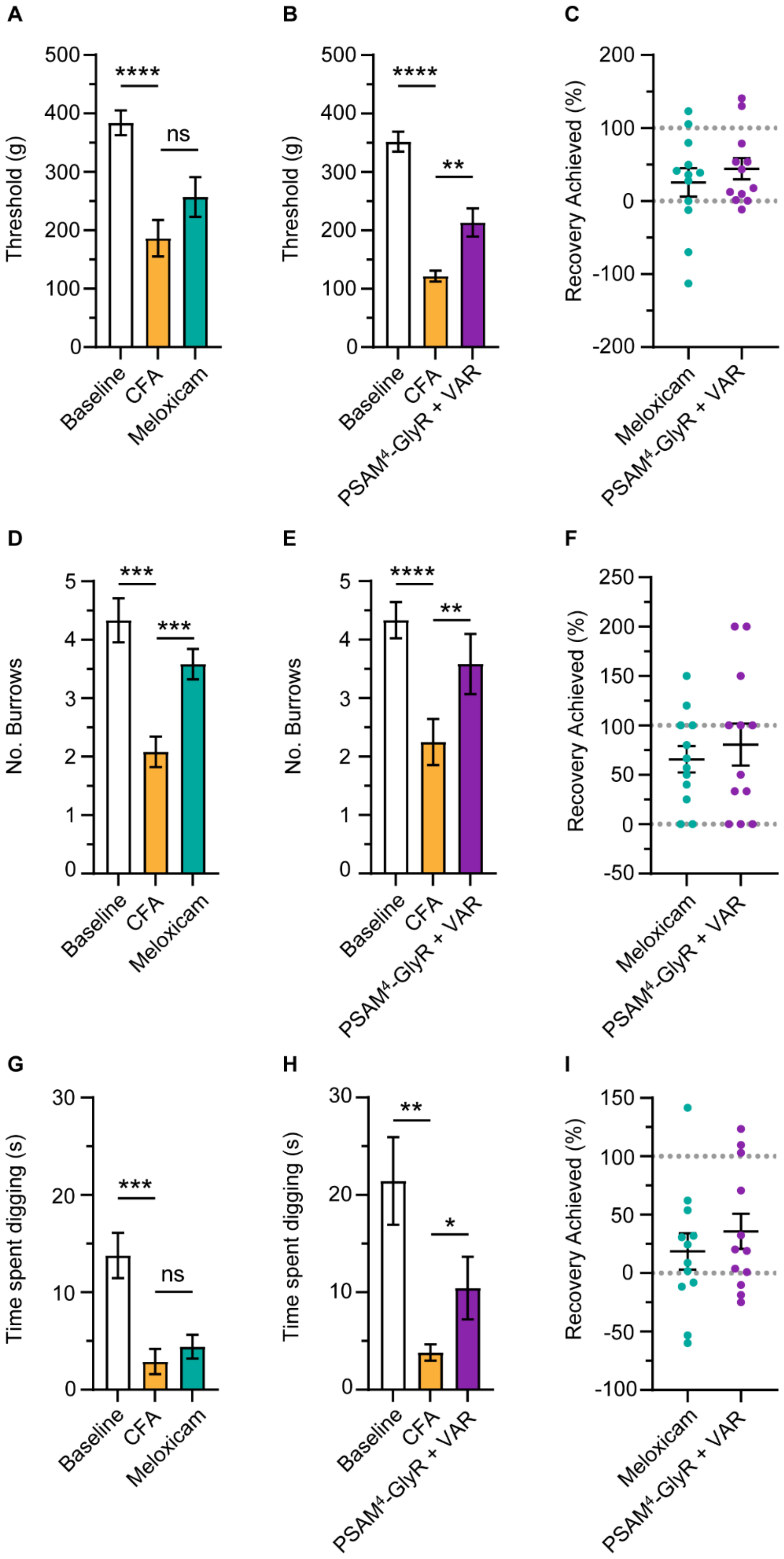
A
500
400
300
200
100
0
Threshold (g)
****
ns
Baseline
CFA
Meloxicam
B
500
400
300
200
100
0
Threshold (g)
****
**
Baseline
CFA
PSAM4-GlyR + VAR
C
200
100
0
-100
-200
Recovery Achieved (%)
Meloxicam
PSAM4-GlyR + VAR
D
5
4
3
2
1
0
No. Burrows
***
***
Baseline
CFA
Meloxicam
E
5
4
3
2
1
0
No. Burrows
****
**
Baseline
CFA
PSAM4-GlyR + VAR
F
250
200
150
100
50
0
-50
Recovery Achieved (%)
Meloxicam
PSAM4-GlyR + VAR
G
30
20
10
0
Time spent digging (s)
***
ns
Baseline
CFA
Meloxicam
H
30
20
10
0
Time spent digging (s)
**
*
Baseline
CFA
PSAM4-GlyR + VAR
I
150
100
50
0
-50
-100
Recovery Achieved (%)
Meloxicam
PSAM4-GlyR + VAR

Fig. S9: PSAM^4-GlyR inhibition of knee inflammatory-like pain is comparable to Meloxicam.

(A-B) The knee pressure thresholds at baseline, following CFA, and following Meloxicam in wildtype mice (**A**), or following varenlicine in mice expressing PSAM^4-GlyR in sensory neurons (**B**). (**C**) The recovery achieved (methods for details) for Meloxicam, and PSAM^4-GlyR + Varenicline cohorts. **(D-E)** The number of burrows dug at baseline, following CFA, and following Meloxicam in wildtype mice (**D**), or following Varenlicine in mice expressing PSAM4-GlyR in sensory neurons (**E**). (**F**) The recovery achieved for Meloxicam, and PSAM^4-GlyR + varenicline cohorts. **(G-H)** The time spent digging at baseline, following CFA, and following Meloxicam in wildtype mice (**G**), or following varenlicine in mice expressing PSAM4-GlyR in sensory neurons (**H**). (**I**) The recovery achieved for Meloxicam, and PSAM^4-GlyR + varenicline cohorts. 0 = no recovery, 100 = full recovery. A, B, D, E, G, H: n = 12 mice, RM-one way ANOVA, post-hoc Holm-Šídák's tests compared to CFA, n.s. P > 0.05, * P < 0.05, ** P < 0.01, *** P < 0.001, **** P < 0.0001. C, F, I: n = 12 mice, unpaired t-test, P > 0.05). Data mean ± S.E.M.

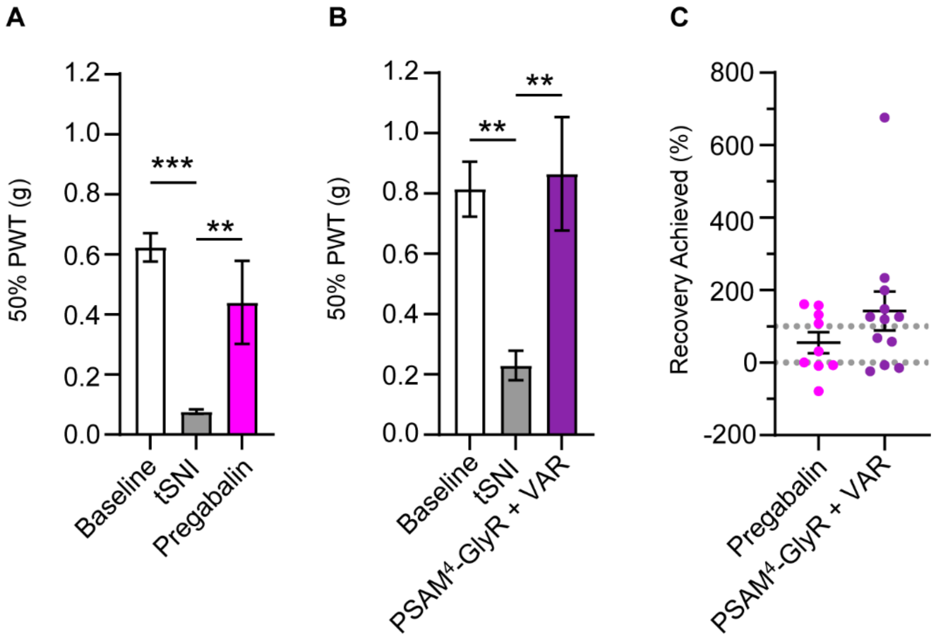

Fig. S10: PSAM^4-GlyR inhibition of neuropathic-like pain is comparable to Pregabalin.

(A) The paw withdrawal threshold of wildtype mice at baseline, 7 days following tibial spared nerve injury (tSNI), and 14 days following tSNI after Pregabalin treatment (n = 9 mice, RM-one way ANOVA, post-hoc Holm-Šídák's tests compared to SNI, ** P < 0.01, *** P < 0.001). **(B)** The paw withdrawal threshold of mice (that previously received i.t. AAV9-PSAM^4-GlyR) at baseline, 7 days following tSNI, and 14 days following tSNI after varenicline treatment (n = 12 mice, RM-one way ANOVA, post-hoc Holm-Šídák's tests compared to SNI, ** P < 0.01). **(C)** The recovery achieved for Pregabalin and PSAM^4-GlyR + varenicline cohorts. 0 = no recovery, 100 = full recovery. (Pregabalin n = 9, PSAM^4-GlyR + Var n = 12, Mann Whitney t-test, P > 0.05). Data mean ± S.E.M.

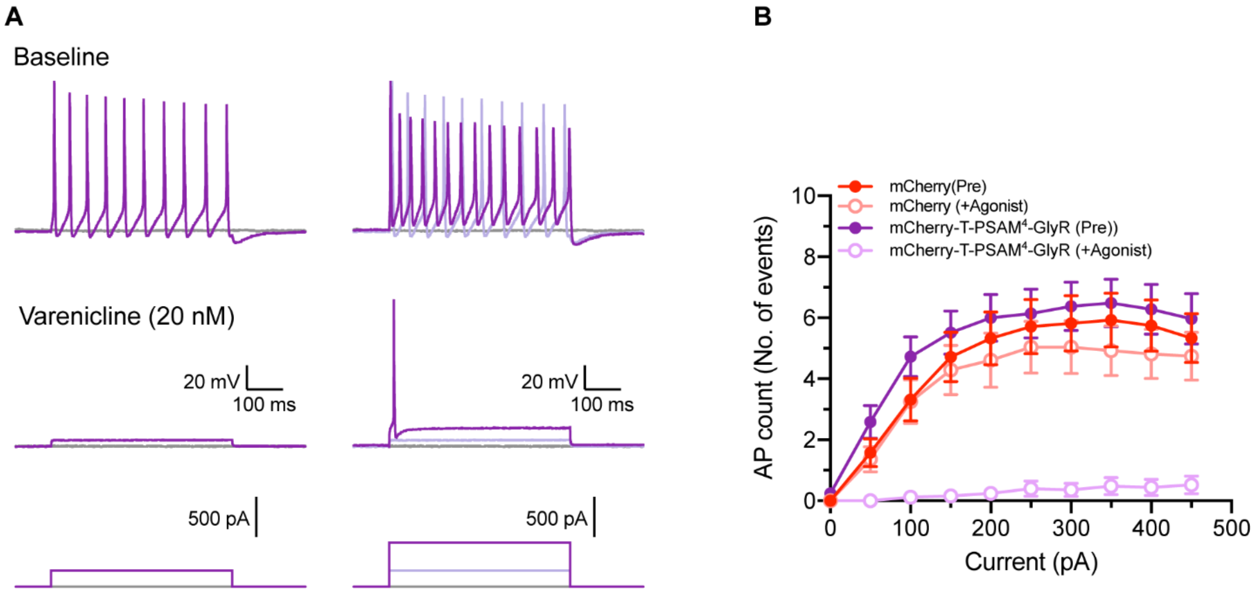

Fig. S11. Repetitive firing of human-derived sensory neurons is abolished by PSAM^4-GlyR activation.

(A) Example traces showing repetitive firing with increasing current application before activation of PSAM^4-GlyR (Top trace). After varenicline application, repetitive firing in PSAM^4-GlyR expressing neurons was abolished. Some neurons fired a single AP upon application of a large current pulse (Bottom trace). **(B)** Quantification of the number of APs with increasing current pulses. Recordings in the presence of uPSEM^{792} and varenicline were pooled and labeled as +Agonist. Data expressed as mean ± S.E.M.

Supplementary Tables

Table S1. Fragments and digestion enzymes used to generate mCherry-T-PSAM^4-GlyR.

No.	Name	5’ restriction site	3’ restriction site	Source
1	Kozak-(partial)mCherry	NheI	SbfI	Twist Bioscience
2	(Partial)mCherry	SbfI	SgrAI	Twist Bioscience
3	Tandem-(partial)PSAM4	SgrAI	SexAI	Twist Bioscience
4	(Partial)PSAM4-GlyR	SexAI	AgeI	PCR from #119739*

* AgeI was introduced using a reverse primer overhang

Table S2. Antibodies used in this study.

**Primary Antibody**	**Source**	**Identifier**
NeuN (1:500, Rabbit)	Abcam	Ab177487
NeuN (1:500, Chicken)	Merck Millipore	Abn91
IB4 (1:50, Streptavidin conjugated)	Sigma-Aldrich	L2140
CGRP (1:250, Sheep)	Enzo	Ca1137
NF200 (1:1000, Rabbit)	Merck Millipore	ABN76
Parvalbumin (1:200, Guinea pig)	Frontier Institute	Af1000
Tyrosine Hydroxylase (1:250, Sheep)	Merck Millipore	Ab1542
ATF3 (1:300, Rabbit)	Novus Bio	NBP1-85816
Beta III tubulin (1:1,000, Rabbit)	Abcam	Ab18207
**Secondary Antibody**	**Source**	**Identifier**
Pacific Blue (1:100)	Thermo Fischer Scientific	Alexa Fluor
Alexa Fluor 488 (1:500)	Thermo Fischer Scientific	Alexa Fluor
Alexa Fluor 546 (1:500)	Thermo Fischer Scientific	Alexa Fluor
